# Supplementary figures and images for: Single-cell differential expression analysis between conditions within nested settings
Source: Brief Bioinform. 2025 Aug 12;26(4):bbaf397. doi: 10.1093/bib/bbaf397 (PMC12343076; doi:10.1093/bib/bbaf397)

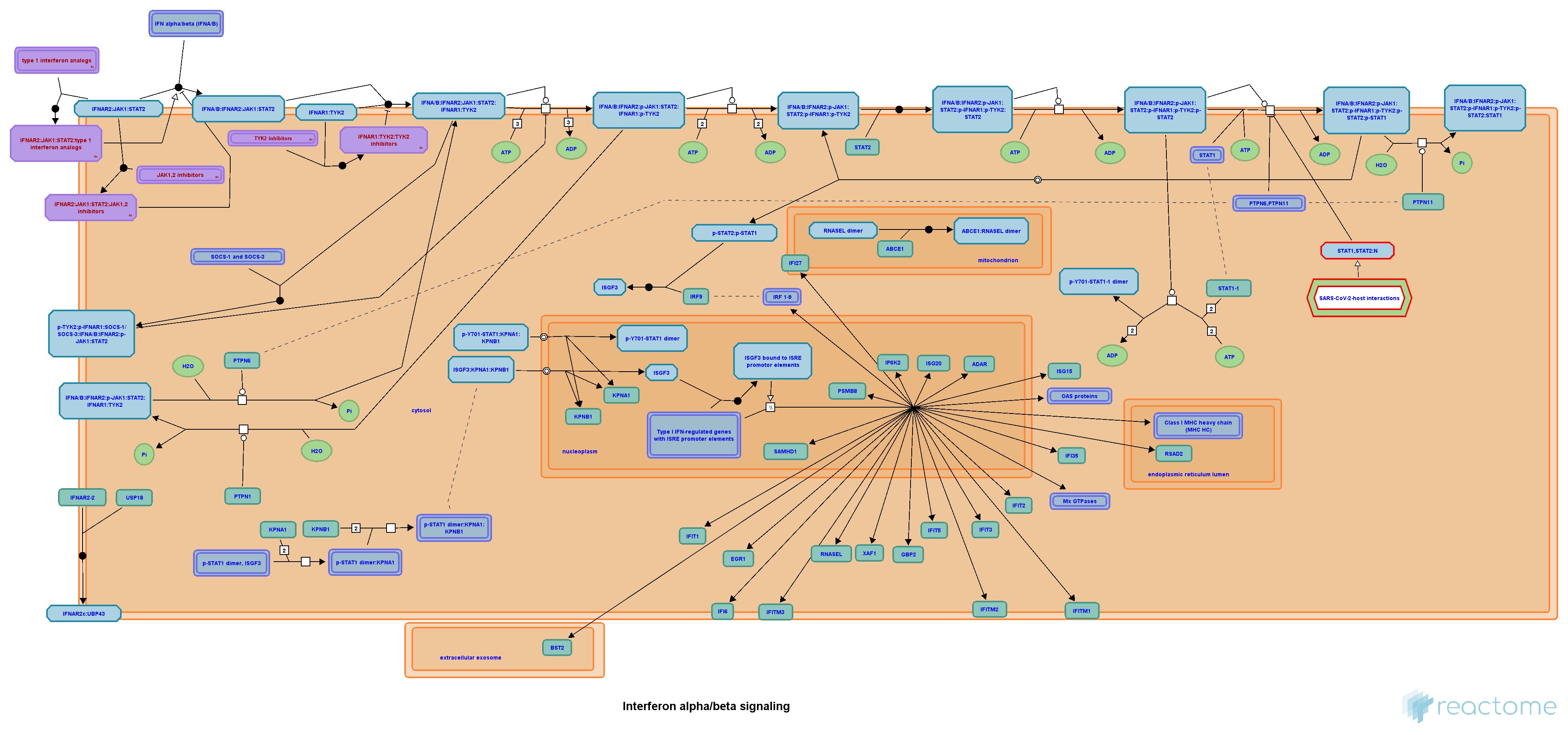

Supplement: Fig_S01_bbaf397 [file fig_s01_bbaf397.jpeg]

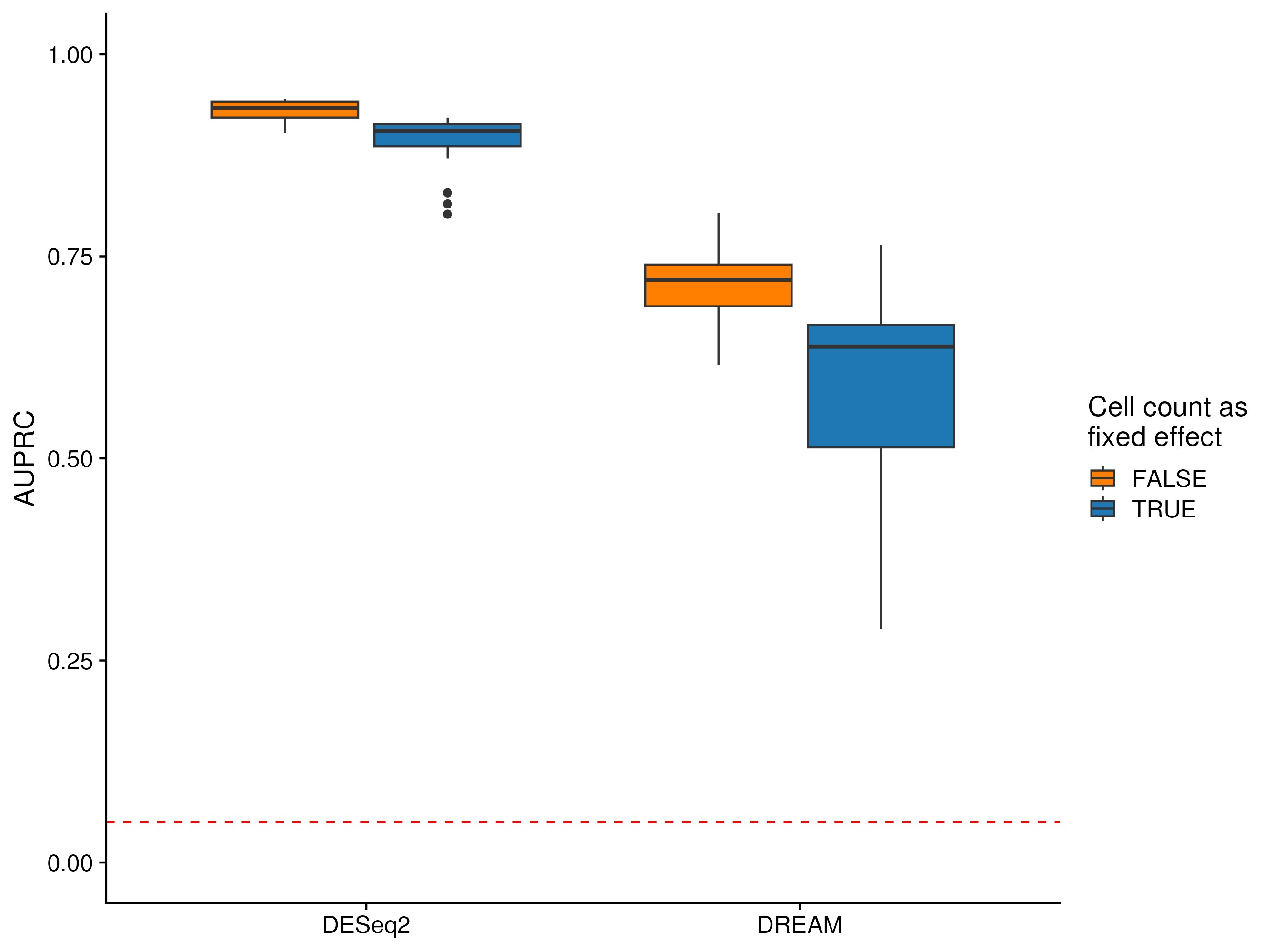

Supplement: Fig_S02_bbaf397 [file fig_s02_bbaf397.jpeg]

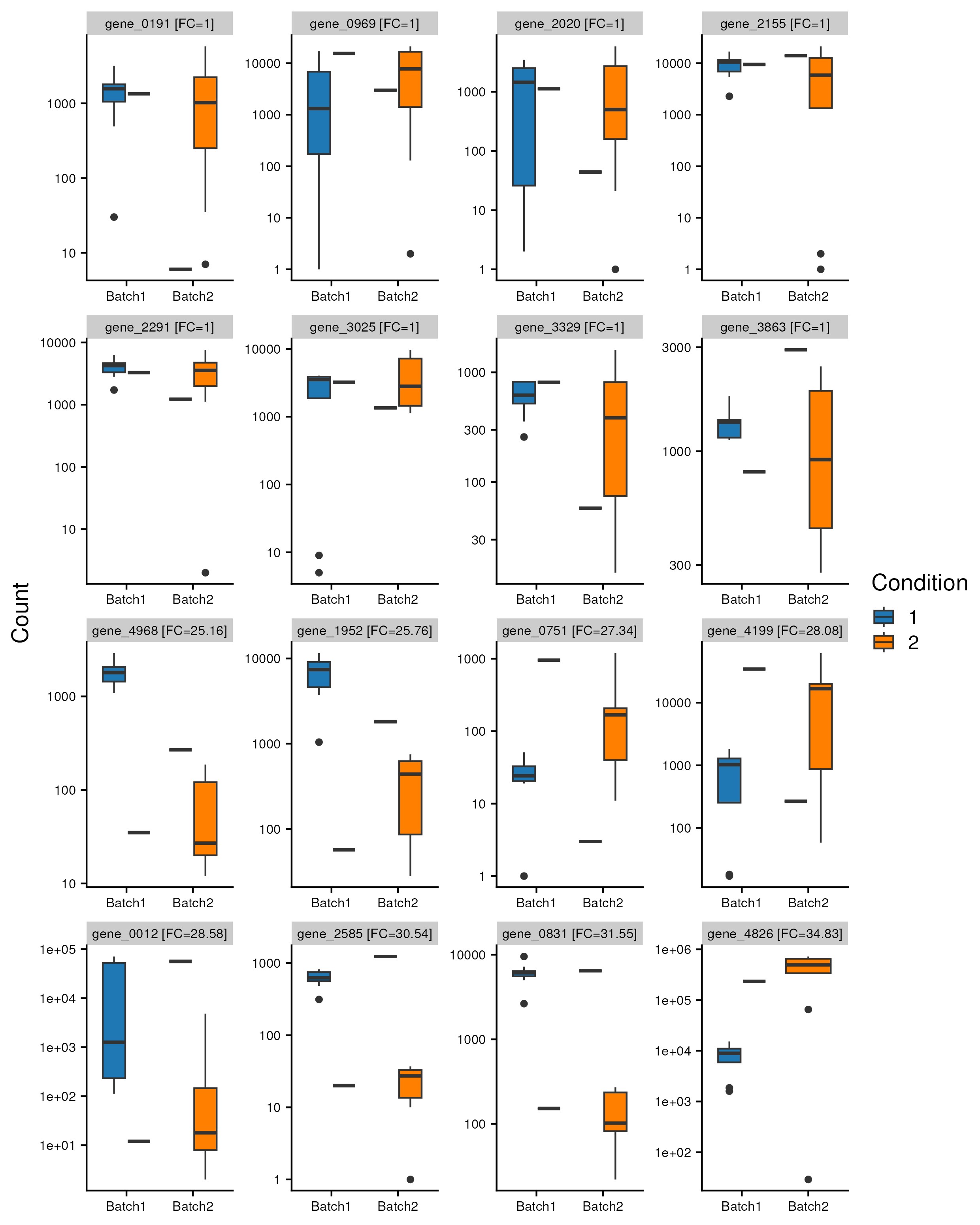

Supplement: Fig_S03_run1_bbaf397 [file fig_s03_run1_bbaf397.jpeg]

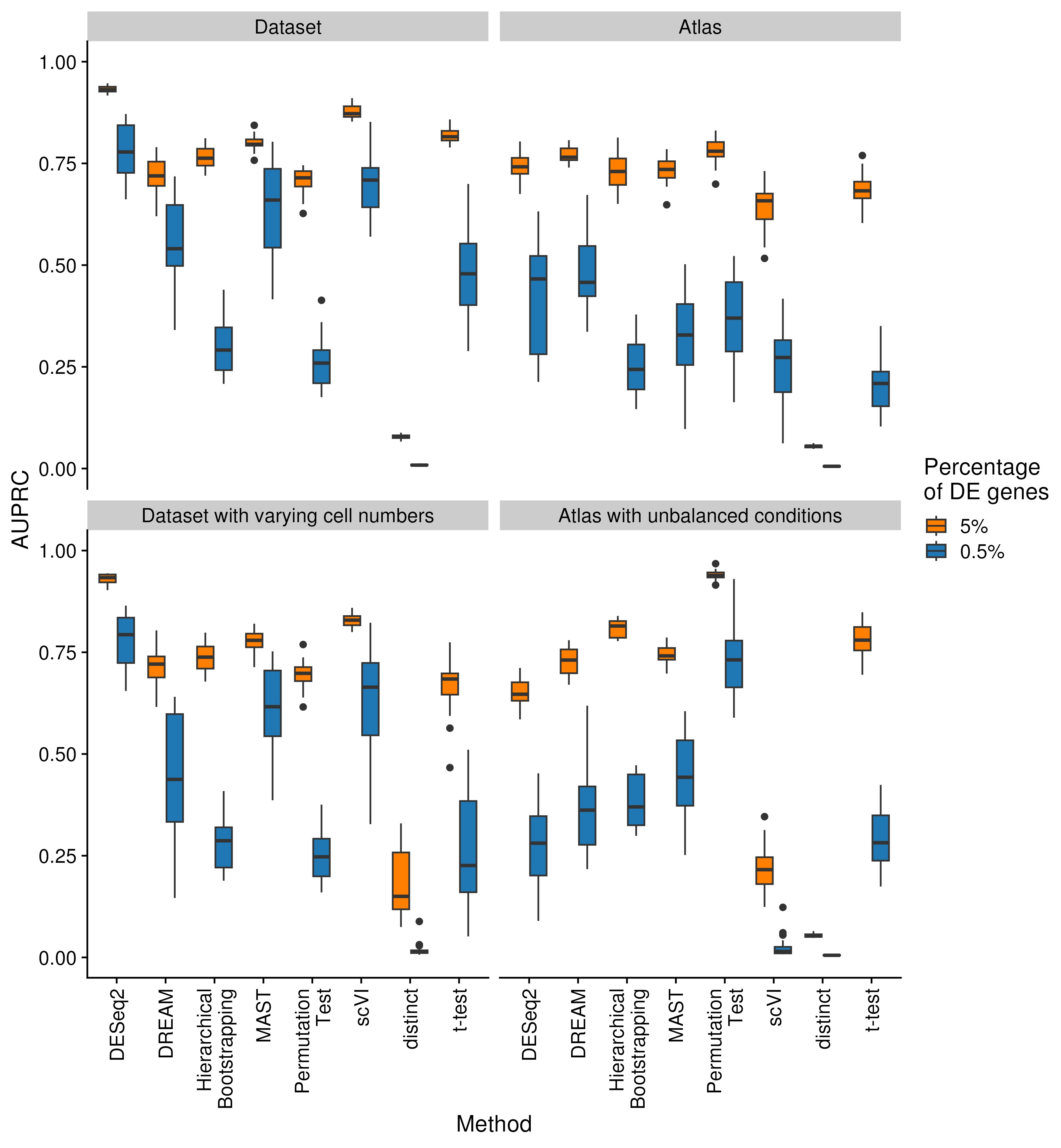

Supplement: Fig_S04_bbaf397 [file fig_s04_bbaf397.jpeg]

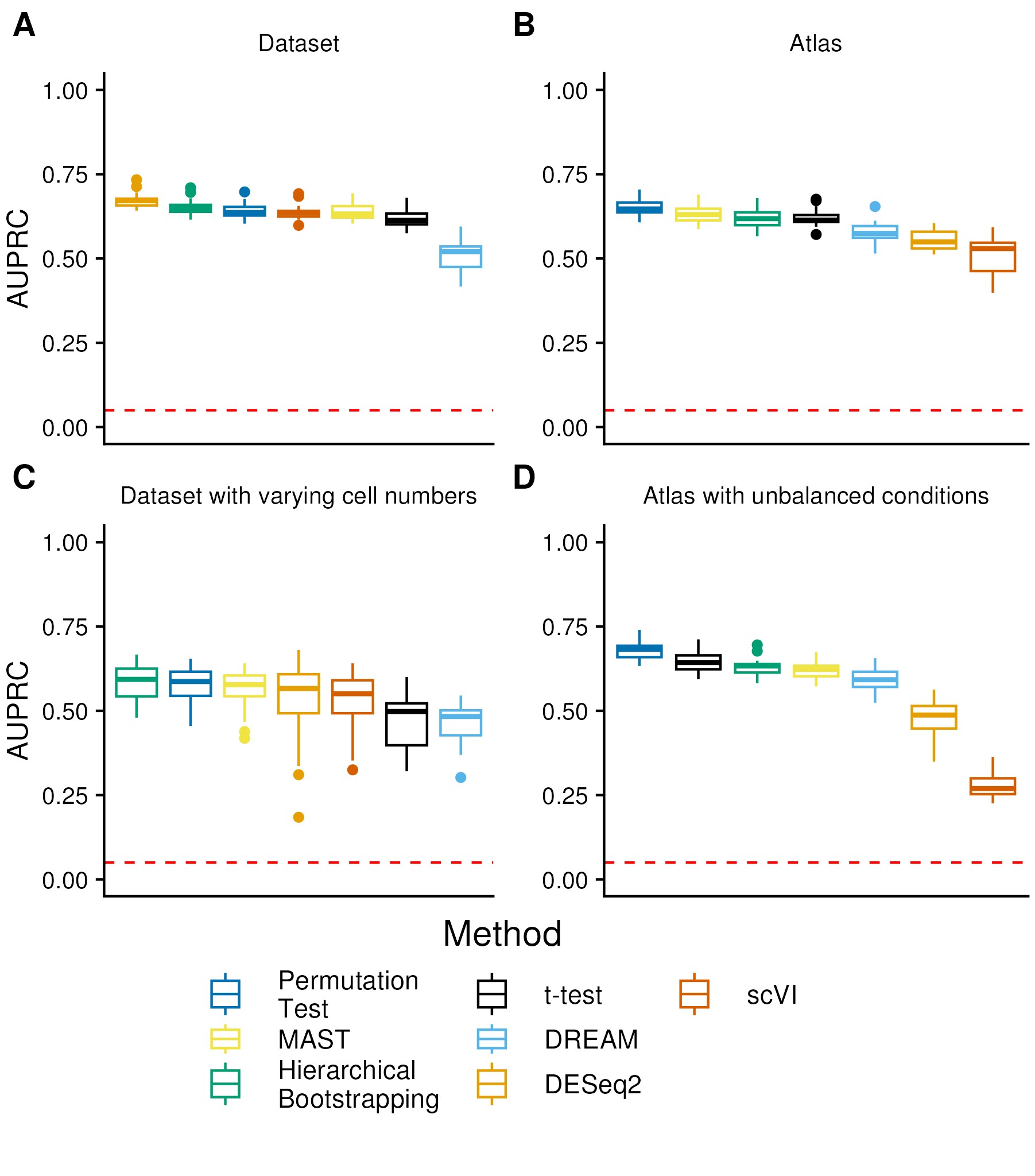

Supplement: Fig_S05_bbaf397 [file fig_s05_bbaf397.jpeg]

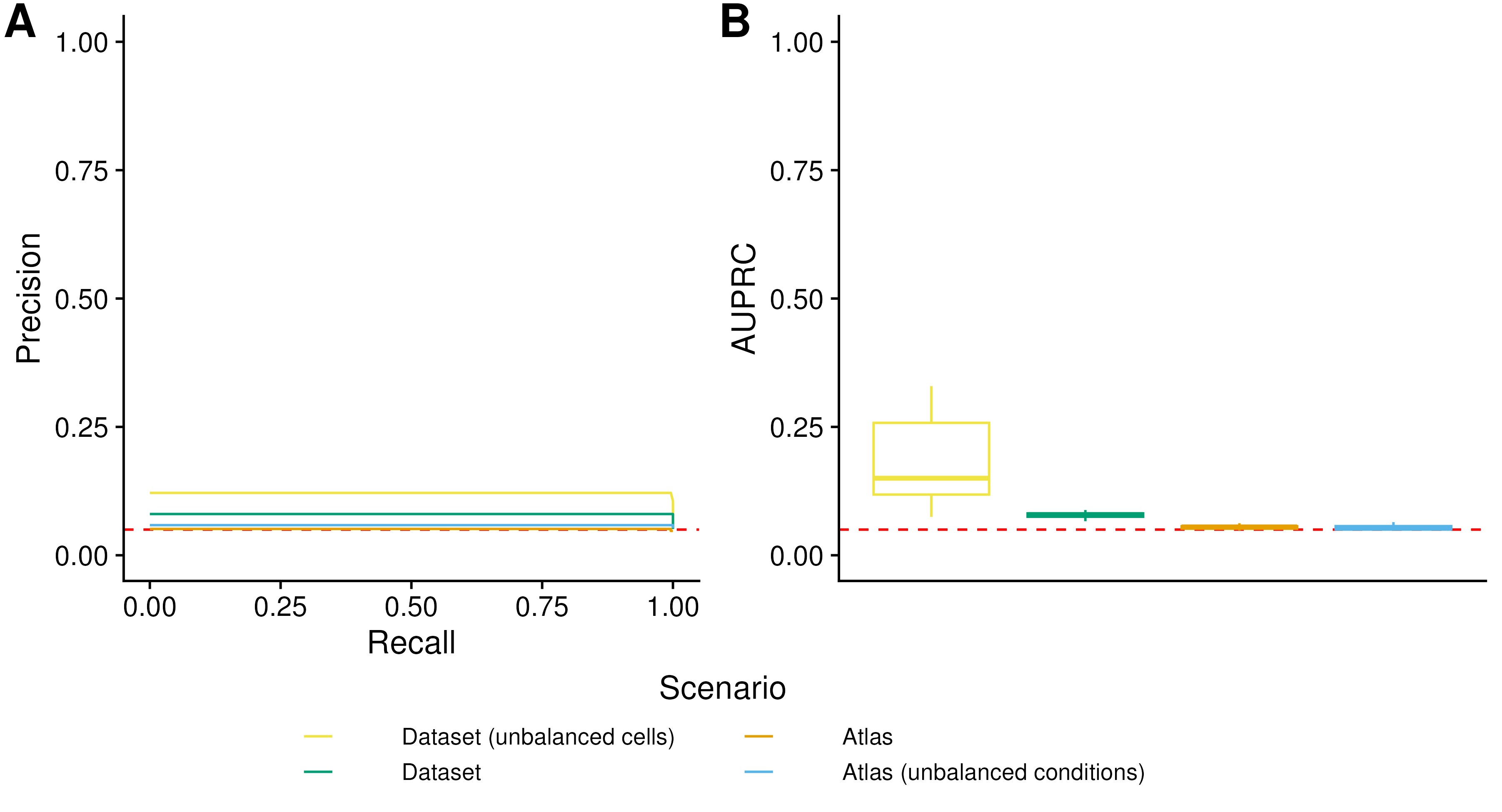

Supplement: Fig_S06_run1_bbaf397 [file fig_s06_run1_bbaf397.jpeg]

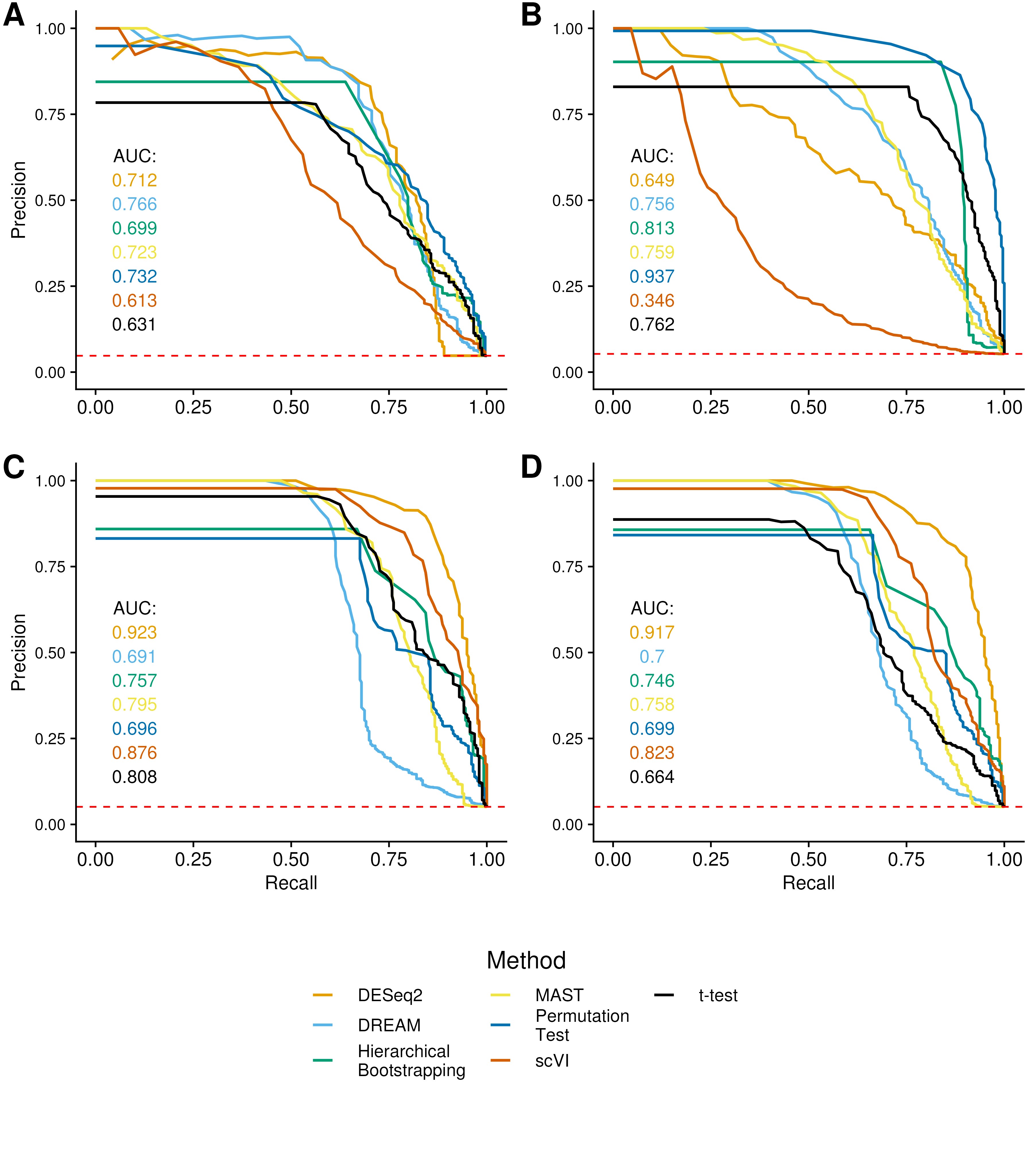

Supplement: Fig_S07_run1_bbaf397 [file fig_s07_run1_bbaf397.jpeg]

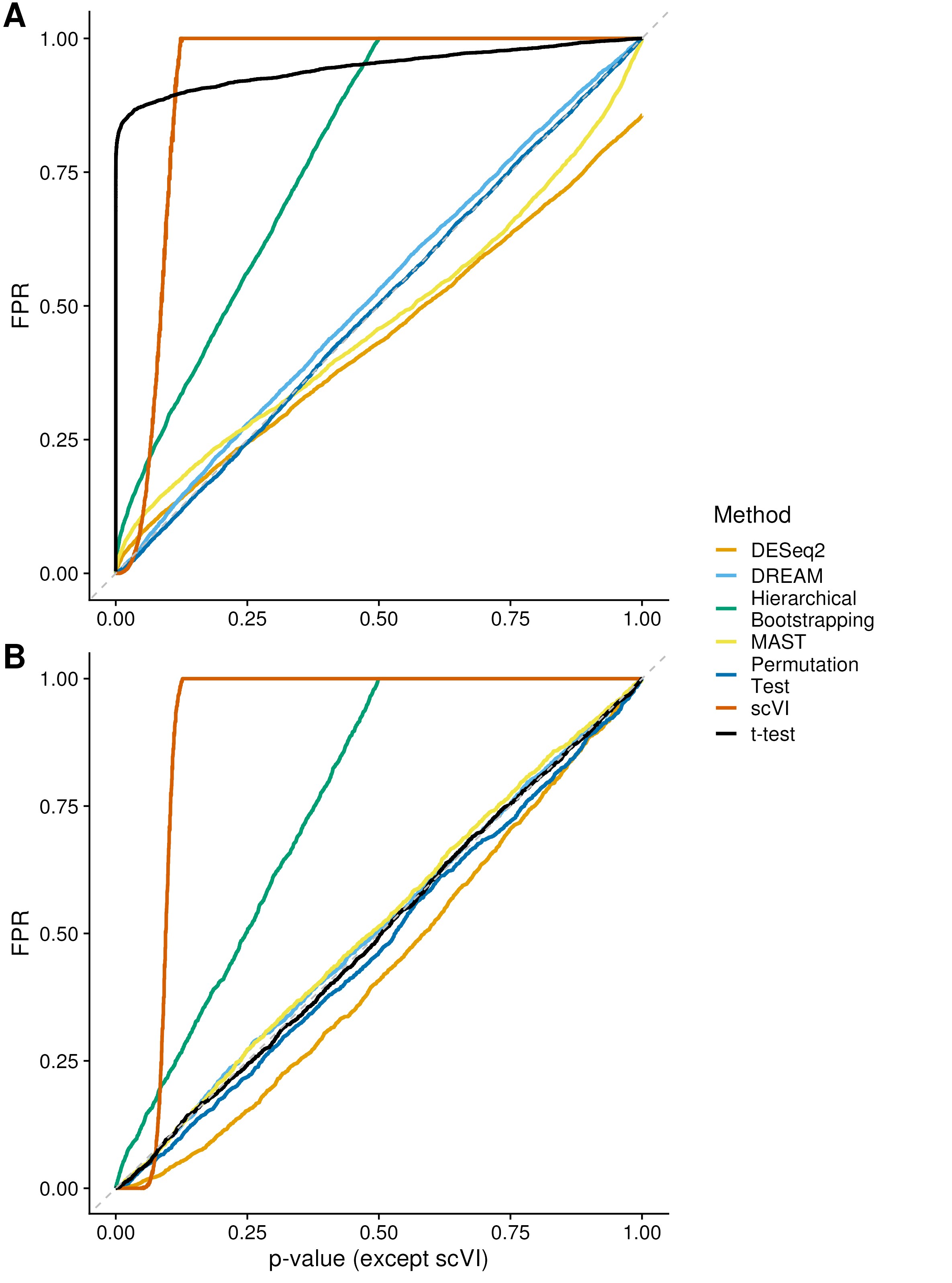

Supplement: Fig_S08_bbaf397 [file fig_s08_bbaf397.jpeg]

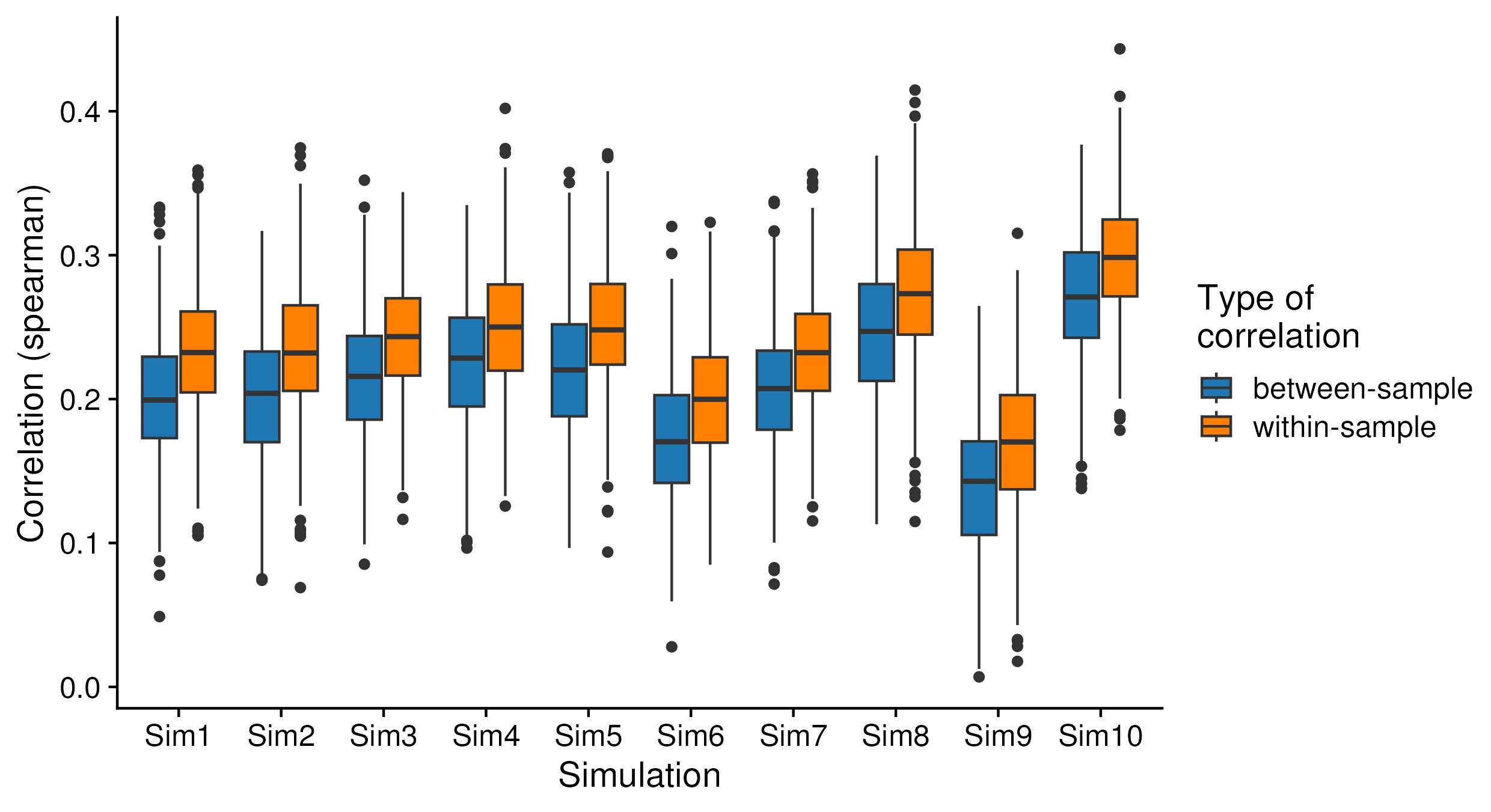

Supplement: Fig_S09_bbaf397 [file fig_s09_bbaf397.jpeg]

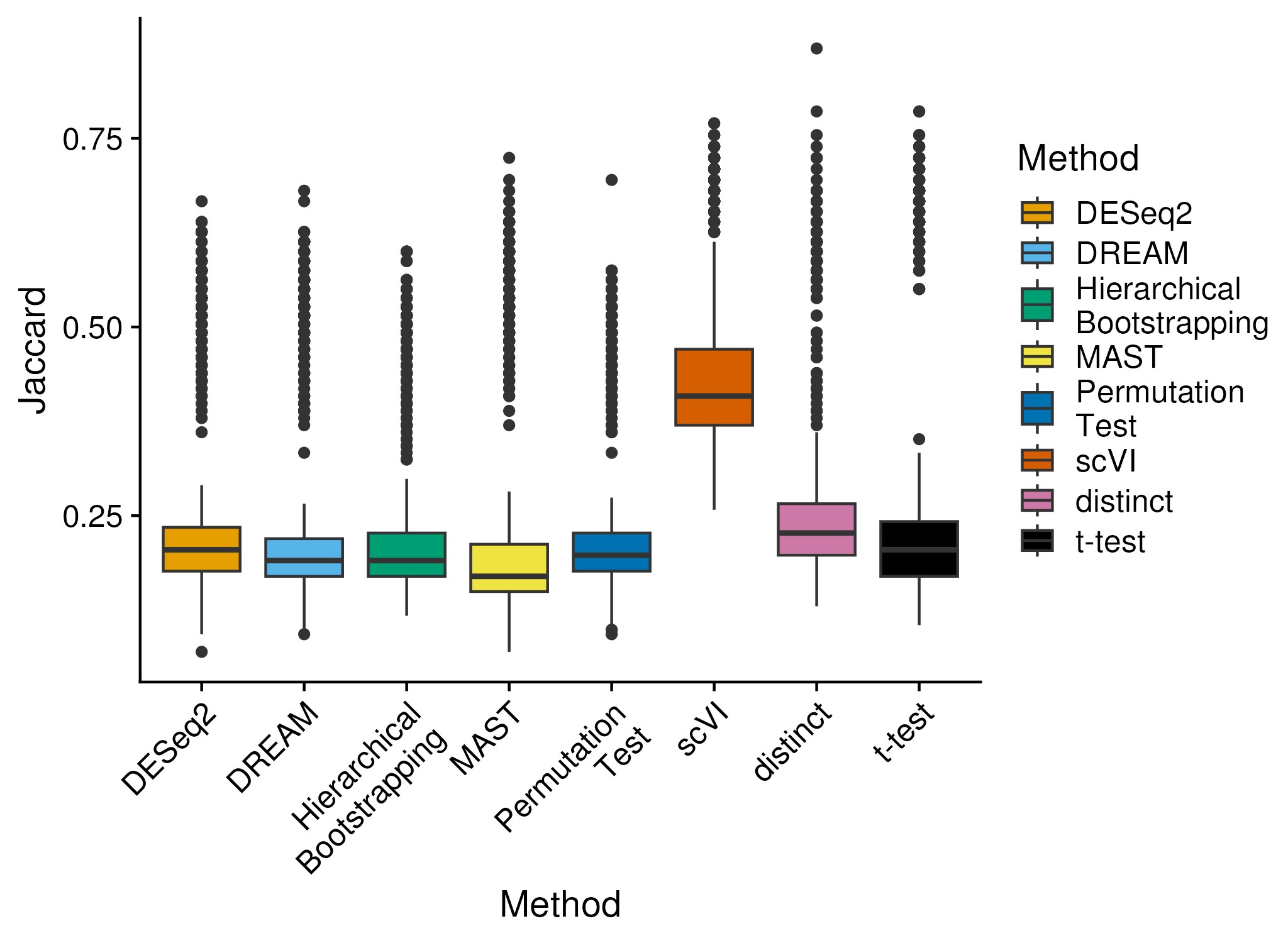

Supplement: Fig_S10_bbaf397 [file fig_s10_bbaf397.jpeg]

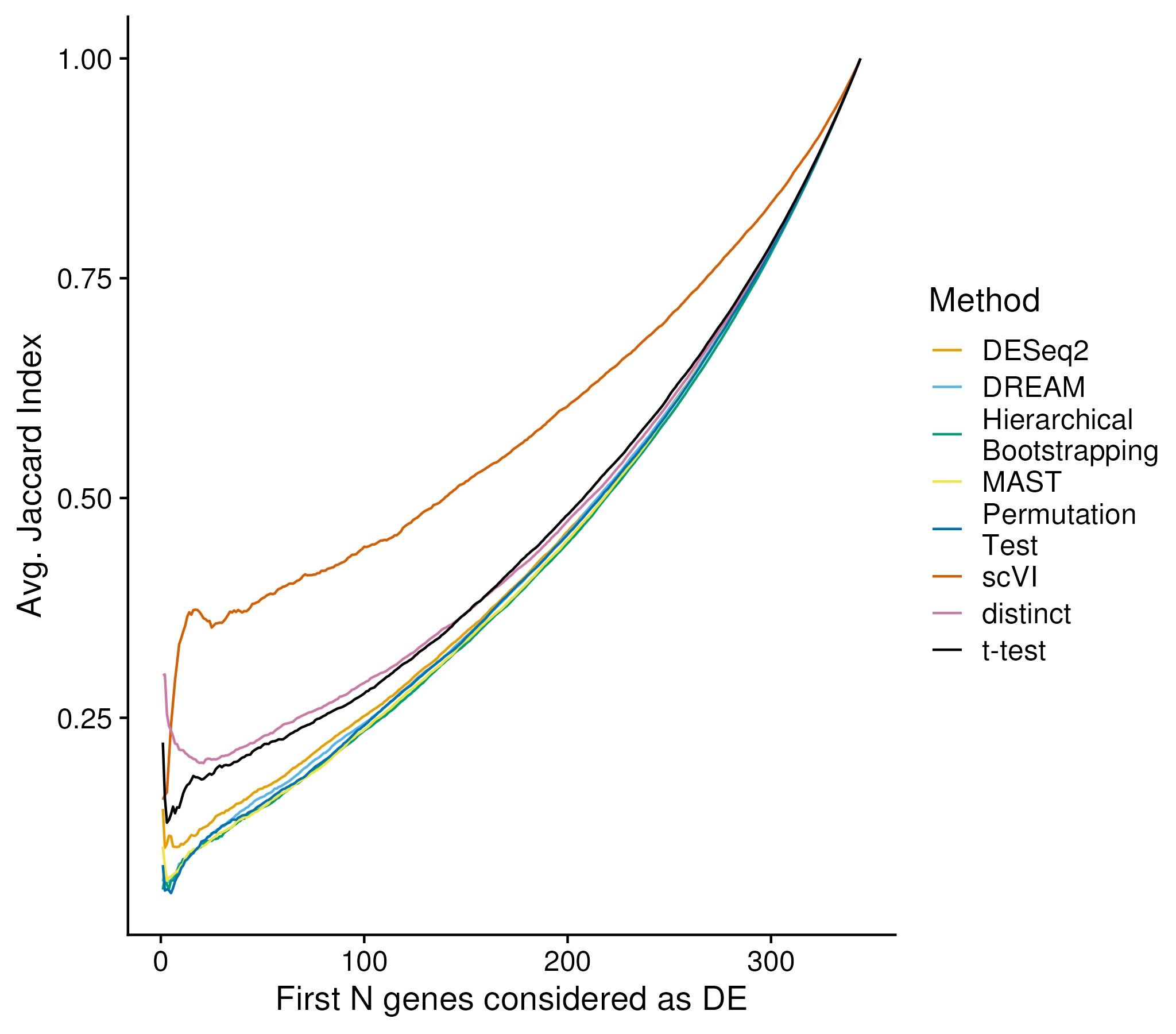

Supplement: Fig_S11_bbaf397 [file fig_s11_bbaf397.jpeg]

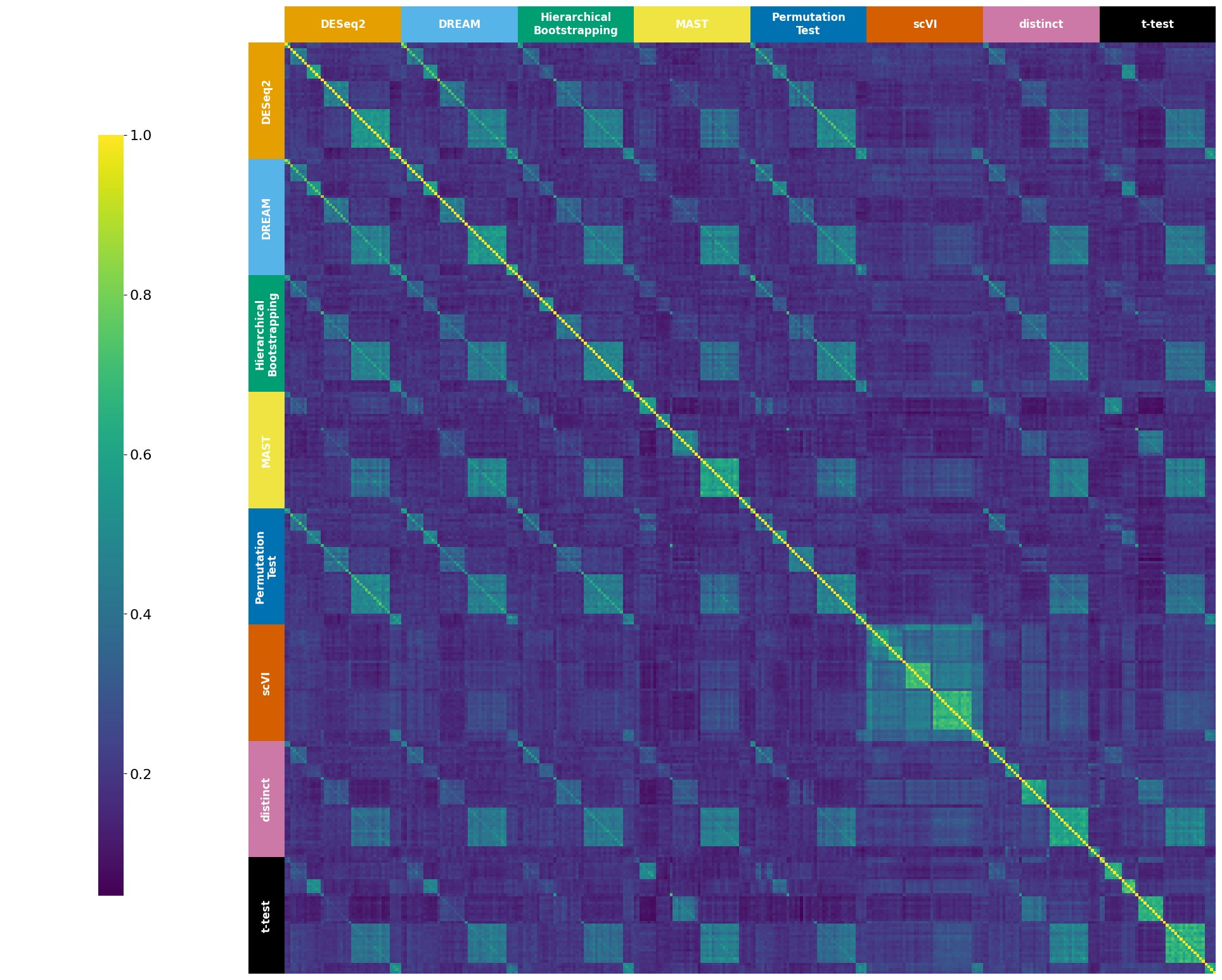

Supplement: Fig_S12_bbaf397 [file fig_s12_bbaf397.jpeg]
