# Supplementary material for: Single-cell differential expression analysis between conditions within nested settings
Source: Brief Bioinform. 2025 Aug 12;26(4):bbaf397. doi: 10.1093/bib/bbaf397 (PMC12343076; doi:10.1093/bib/bbaf397)
Supplement: revision2_supplement_bbaf397 [file revision2_supplement_bbaf397.docx]

**Supplement: Single-cell differential expression analysis between conditions within nested settings**

**Supplementary Note 1: Method Implementations**

**DESeq2**

The raw simulation data without normalization was used as input. The design matrix for DESeq2 varied among the different scenarios. For the Atlas scenarios and also on the real data, batch and condition information were included:

~ Batch + Condition

For the Dataset scenarios, only the information about the condition was provided:

~ Condition

We also assessed the method's performance on the Dataset with varying cell numbers per sample by including the logarithmized number of cells per pseudobulk (PB) as a fixed effect in the design matrix:

~ log(#Cells per PB + Condition)

In the first step, a DESeq2 dataset based on the counts was created. Size factors were calculated prior to differential expression analysis. We used R version 4.2.3, DESeq2 1.38.3 and anndataR 0.99.0, to transfer the gene expression data between Python and R.

**Permutation Test**

The Permutation Test was implemented using counts per million (cpm) normalized single-cell count data as input. Our implementation allows users to specify the minimum and maximum number of iterations (10000 and 100000 by default) as parameters. We permuted the condition labels of all cells 10000 times and compared these permutations to the initial condition assignment. If none of these permutations resulted in an assignment more extreme than the initial one, we adaptively increased the number of iterations to 100000, seeking at least one more extreme permutation. Should no permutation be more extreme than the initial assignment, the method outputs the p-value 0, which indicates a value below 1e-5. We used Python 3.11.9, Scanpy 1.10.1, Anndata 0.10.7, Pandas 2.2.2 and NumPy 1.26.4 to process the data and implement the Permutation Test.

**distinct**

The data preprocessing adhered to the guidelines provided by the developer of the distinct package. Log-normalized counts and a design matrix were used as inputs for the primary function of distinct. The design matrix included batch and condition assignments for the Atlas and the real scenario; only the condition assignment was used for the Dataset scenarios. The globally adjusted p-values derived from the distinct output were propagated throughout the analysis pipeline. We conducted benchmarks using distinct 1.10.0. To prepare log-normalized counts, we utilized R 4.2.3, scuttle 1.8.4 and SingleCellExperiment 1.20.1. Additionally, anndataR 0.99.0 facilitated the conversion of the data into R objects.

**DREAM**

DREAM (Differential expression for repeated measures) is a method for differential expression testing on bulk RNA-Seq data and is therefore applied to pseudobulked single-cell data.

The expression data processing mirrored the approach outlined in the DREAM vignette. A DGEList object was created from the raw counts, from which normalizing factors were derived. In the case of the Atlas scenarios, a random effect was modeled for the batches as follows:

~ Condition + (1 | Batch)

For the dataset scenarios, only the condition was modeled:

~ Condition

For the Dataset with varying cell numbers per sample we included the logarithmized number of cells per pseudobulk as an additional factor into the design matrix:

~ Condition + log(#Cells per PB)

Due to pseudobulking, there was no need to model the sample level separately, as each sample and gene combination yielded a single expression value. The voomWithDreamWeights function was used to generate a DREAM object, and the data analysis was conducted using the dream and eBayes functions. Limma was finally used to extract the differentially expressed genes.

The p-value produced in the output table was used for further analyses.

For benchmarking, we used R 4.2.3 and the variancePartition package version 1.28.9.

The data processing relied on data.table 1.14.8, edgeR 3.40.2 and SingleCellExperiment 1.20.1. AnndataR 0.99.0 was employed to transfer the data from Python to R.

**MAST**

MAST received log-transformed counts as input. For the dataset scenarios, sample information was included and modeled as a random effect. This modeling approach is analogous to pseudobulking and counteracts the pseudoreplication bias. This results in the following model:

~ Condition + (1 | Sample)

The batch effects of the atlases were modeled as a random effect:

~ Condition + (1 | Batch) + (1 | Sample)

While these could theoretically be modeled as fixed effects, that would require that each batch includes at least one sample from each condition to be valid.

After fitting the model, the hurdle model's p-value and logarithmic fold changes were extracted for the condition coefficient. R 4.2.3 and MAST 1.24.1 were used. AnndataR 0.99.0 facilitated the conversion of gene expression objects from Python to R.

**scVI**

ScVI was trained directly on the raw single-cell expression data, following the guidelines outlined in the scVI vignette. The setup_anndata function was used to create an instance of the model. Both Batch and Sample were specified as categorical keys for the atlas scenarios and the real-world dataset. For the dataset scenarios, only the Sample covariate was used. The mini-batch size was dynamically increased if splitting the data resulted in a mini-batch containing only a single gene.

The trained model was evaluated by grouping the data according to condition values.

The probability of a gene not being differentially expressed was treated as the p-value for further analyses, with the exception of the negative control. For the negative control, following the advice of the scVI developers, we counted the differentially expressed genes at 2000 equidistant cutoffs using scVIs "target FDR" parameter and relied on its classification of positives and negatives. The method also adjusts those values to account for multiple testing issues.

Additionally, as the scVI developers also recommend filtering the data for highly variable genes before analysis, we conducted further benchmarks across all methods on datasets that had been filtered to retain only the top 10% of genes with the highest variability. Genes simulated as differentially expressed that were excluded by this filtering process were assessed as false negatives. For this filtering process, the implementation of Seurat_v3 in Scanpy was employed. The benchmark utilized Python 3.11.9 and scVI 1.1.2. Data preprocessing was performed using pandas 2.2.2 and scanpy 1.10.1.

**Hierarchical Bootstrapping**

We implemented Hierarchical Bootstrapping in Python. The cpm normalized single-cell counts serve as input for the method. Additionally, one can specify the hierarchy of the data, the sample size and the aggregation function. The bootstrapping step and the evaluation step are encapsulated in two separate functions. The first one generates samples according to the specified hierarchies and sample sizes, aggregates them and stores them in a new Anndata object. The evaluation step iterates over this object and counts how often the first condition generated higher expression values than the second condition and vice versa. The gene-wise p-value is obtained by the following formula:

$\frac{min(\#(Cond_{1} > Cond_{2}), \#(Cond_{1} < Cond_{2}))}{\#permutations}$

We used Python 3.11.9, Scanpy 1.10.1, Anndata 0.10.7, Pandas 2.2.2 and NumPy 1.26.4 to implement the Hierarchical Bootstrapping method.

**t-test**

The t-test was implemented using the rank_genes_groups function from the Scanpy library. The function was executed with the parameters groupby="Condition" and method="t-test". This method compares the mean expression values between the groups specified in the groupby parameter using a two-sided t-test. All genes were tested individually, and the resulting p-values were used to rank the genes by their differential expression significance.

The implementation adhered to the computational environment used for the other methods implemented in python, which utilized Python 3.11.9, Scanpy 1.10.1, Anndata 0.10.7, and Pandas 2.2.2.

**Supplementary Note 2: Correlation Analysis**

Consistent with the notion of pseudoreplication bias, transcriptome profiles of cells within a sample show higher pairwise correlation compared to two cells from different samples [1]. To confirm that our simulated data showed this behavior, we simulated 10 samples with 250 cells each in a single batch. In this analysis, we first excluded redundant genes with more than 0.1 absolute Spearman correlation and then randomly sampled 500 genes for further analysis. One cell was drawn from each sample, and then the pairwise correlations of the drawn cells were computed to calculate the between-sample correlations. This procedure was repeated 1,000 times, resulting in a total of 45,000 between-sample correlations. For the within-sample correlations, we sampled 4,500 cell pairs (without replacement) within each sample, and computed their Spearman correlations to obtain a total of 45,000 within-sample correlations. This resulted in the within- and between-sample correlations shown in Fig. S9.

The correlation of cells within a sample for each dataset is higher than the correlation across samples. Thus, it can be concluded that cells in the simulated data are not statistically independent and would introduce pseudoreplication bias if not accounted for. This is consistent with the evidence found for real datasets

**Supplementary Note 3. Pseudocode for the Hierarchical Bootstrapping method on single-cell data.**

The first part of the pseudocode describes the bootstrapping step, where we cycle through the number of bootstrapping iterations and draw sample cells based on the specified hierarchy. The second part is called the evaluation step and iterates over the data aggregated in the bootstrapping step. It generated the p-value of a gene by counting in which of the conditions it has a higher expression more frequently.

| *// First step: Bootstrapping phase* output = {} **for** i = 1 **to** n **do**  **for** each condition **do**  resample first hierarchy level with sample size = \|{hierarchy level 1}\|  restrict the selection to the drawn samples  **for** each of the remaining hierarchy levels **do**  resample hierarchy level with user specified sample size  restrict the selection to the drawn samples  **end**  aggregate selected samples with specified aggregation function  add results to output  **end** **end**  *// Second step: Evaluation phase* data := output pvalue = {} **for** each gene **do**  condition1_higher = 0  condition2_higher = 0  **for** i = 1 to n **do**  **if** data[i, condition1] > data[i, condition2] **then**  condition1_higher += 1  **else**  condition2_higher += 1  **end**  **end**  pvalue[gene] = min(condition1_higher / n, condition2_higher / n) **end** |
| --- |

*Table S1. Results of runtime benchmarking with varying number of genes. The runtimes (in seconds) of the methods MAST, distinct, DESeq2, Permutation Test, Hierarchical Bootstrapping (hb), scVI, DREAM, and ttest are shown. In addition, the runtime for simulation and pseudobulking (pb) is depicted. For the runtime graphs, the time required for pseudobulking was added to the runtime of DESeq2, the Permutation Test and DREAM since these require pseudobulked data.*

| **n_genes** | **n_cells** | **sim** | **pb** | **mast** | **distinct** | **deseq2** | **permutation** | **hb** | **scvi** | **dream** | **ttest** |
| --- | --- | --- | --- | --- | --- | --- | --- | --- | --- | --- | --- |
| 100 | 1000 | 29.94 | 4.41 | 54.53 | 74.65 | 20.10 | 174.46 | 358.76 | 43.15 | 18.67 | 27.1 |
| 200 | 1000 | 29.02 | 4.16 | 93.25 | 107.16 | 18.92 | 296.03 | 342.14 | 44.16 | 17.36 | 27.8 |
| 300 | 1000 | 28.59 | 4.10 | 134.14 | 144.15 | 18.98 | 437.50 | 357.94 | 43.93 | 16.46 | 27.2 |
| 400 | 1000 | 30.02 | 4.31 | 184.94 | 189.07 | 20.15 | 580.39 | 396.54 | 53.45 | 17.36 | 26.1 |
| 500 | 1000 | 30.51 | 4.22 | 217.28 | 207.65 | 18.82 | 948.75 | 377.58 | 51.74 | 16.80 | 27.1 |
| 600 | 1000 | 30.50 | 4.29 | 268.28 | 242.31 | 19.36 | 854.74 | 399.23 | 54.15 | 16.37 | 27.6 |
| 700 | 1000 | 31.23 | 4.47 | 309.87 | 289.04 | 20.08 | 1156.48 | 432.90 | 62.00 | 17.08 | 26.8 |
| 800 | 1000 | 31.05 | 4.26 | 337.23 | 296.56 | 19.28 | 1325.13 | 417.50 | 53.93 | 16.26 | 27.0 |
| 900 | 1000 | 30.92 | 4.12 | 381.33 | 331.39 | 19.01 | 1521.79 | 434.53 | 56.34 | 16.10 | 27.7 |
| 1000 | 1000 | 32.84 | 4.59 | 425.66 | 372.78 | 21.01 | 1373.66 | 487.07 | 63.90 | 16.99 | 27.1 |
| 1500 | 1000 | 32.27 | 4.10 | 594.35 | 496.48 | 19.09 | 2117.92 | 532.59 | 64.05 | 16.16 | 26.4 |
| 2000 | 1000 | 35.50 | 4.49 | 812.80 | 662.02 | 20.95 | 3035.82 | 726.20 | 78.41 | 17.55 | 28.5 |
| 2500 | 1000 | 33.81 | 4.08 | 953.51 | 721.92 | 19.88 | 3585.70 | 777.25 | 75.60 | 16.01 | 26.5 |
| 3000 | 1000 | 37.24 | 4.47 | 1218.05 | 870.54 | 21.27 | 4660.22 | 1033.40 | 96.77 | 17.75 | 26.5 |
| 3500 | 1000 | 35.60 | 4.10 | 1372.22 | 933.59 | 19.94 | 5320.79 | 1100.95 | 97.82 | 16.29 | 26.3 |
| 4000 | 1000 | 36.93 | 4.17 | 1578.51 | 1066.74 | 20.84 | 6037.05 | 1401.18 | 112.92 | 17.00 | 27.1 |
| 4500 | 1000 | 39.33 | 4.32 | 1740.50 | 1193.55 | 21.07 | 7415.66 | 1622.54 | 121.20 | 17.61 | 27.2 |
| 5000 | 1000 | 36.78 | 4.25 | 1828.64 | 1206.80 | 20.70 | 7816.86 | 1733.96 | 122.72 | 16.83 | 27.4 |
| 5500 | 1000 | 40.87 | 4.64 | 2168.41 | 1408.51 | 22.43 | 8958.25 | 2306.74 | 151.84 | 18.48 | 27.1 |
| 6000 | 1000 | 39.53 | 4.19 | 2218.31 | 1419.10 | 21.46 | 9754.56 | 2376.30 | 141.27 | 17.05 | 27.0 |
| 6500 | 1000 | 41.72 | 4.38 | 2396.07 | 1524.01 | 22.27 | 10693.01 | 2771.10 | 157.94 | 17.28 | 27.4 |
| 7000 | 1000 | 40.74 | 4.24 | 2561.71 | 1603.12 | 21.55 | 11698.24 | 3116.40 | 169.71 | 17.90 | 27.7 |
| 7500 | 1000 | 44.13 | 4.61 | 2818.79 | 1749.01 | 24.28 | 13691.40 | 3716.01 | 197.50 | 18.78 | 26.9 |
| 8000 | 1000 | 43.31 | 4.49 | 2929.63 | 1780.76 | 23.80 | 13736.05 | 3886.08 | 188.50 | 17.82 | 28.5 |
| 8500 | 1000 | 45.04 | 4.23 | 2998.42 | 1797.89 | 23.44 | 15725.48 | 4263.53 | 200.24 | 17.91 | 28.2 |
| 9000 | 1000 | 44.76 | 4.29 | 3145.61 | 1871.13 | 23.17 | 15489.87 | 4658.51 | 208.02 | 17.81 | 29.6 |
| 9500 | 1000 | 43.50 | 4.01 | 3261.59 | 1909.15 | 23.14 | 17352.88 | 5035.23 | 220.77 | 20.64 | 25.3 |
| 10000 | 1000 | 44.43 | 3.97 | 3376.53 | 1960.28 | 23.39 | 17333.21 | 5309.40 | 213.99 | 17.54 | 27.0 |

*Table S2. Results of runtime benchmarking with varying number of cells. The runtimes (in seconds) of the methods MAST, distinct, DESeq2, Permutation Test, Hierarchical Bootstrapping (hb), scVI, DREAM, and ttest are shown. In addition, the runtime for simulation and pseudobulking (pb) is depicted. For the runtime graphs, the time required for pseudobulking was added to the runtime of DESeq2, the Permutation Test and DREAM since these require pseudobulked data.*

| **n_genes** | **n_cells** | **sim** | **pb** | **mast** | **distinct** | **deseq2** | **permutation** | **hb** | **scvi** | **dream** | **ttest** |
| --- | --- | --- | --- | --- | --- | --- | --- | --- | --- | --- | --- |
| 1000 | 100 | 36.91 | 6.86 | 222.38 | 75.43 | 21.05 | 1525.63 | 478.77 | 21.07 | 17.94 | 25.1 |
| 1000 | 200 | 39.02 | 6.53 | 290.65 | 103.28 | 21.00 | 1517.41 | 464.82 | 37.81 | 20.94 | 26.9 |
| 1000 | 300 | 39.11 | 5.27 | 327.33 | 149.41 | 21.16 | 1594.45 | 470.72 | 39.48 | 20.91 | 26.5 |
| 1000 | 400 | 34.96 | 5.39 | 371.92 | 188.67 | 20.35 | 1574.20 | 456.12 | 34.86 | 18.39 | 26.0 |
| 1000 | 500 | 36.51 | 4.67 | 416.09 | 225.08 | 21.95 | 1532.28 | 478.04 | 48.45 | 18.23 | 26.5 |
| 1000 | 600 | 37.65 | 4.72 | 448.13 | 267.46 | 22.66 | 1584.39 | 507.69 | 53.42 | 18.42 | 26.9 |
| 1000 | 700 | 35.33 | 4.61 | 473.68 | 309.27 | 21.36 | 1562.03 | 501.47 | 51.77 | 18.75 | 27.1 |
| 1000 | 800 | 33.30 | 4.43 | 461.20 | 359.04 | 20.45 | 1527.83 | 471.59 | 55.68 | 21.58 | 27.8 |
| 1000 | 900 | 33.82 | 4.49 | 537.26 | 396.85 | 20.28 | 1478.62 | 447.07 | 56.50 | 18.50 | 25.8 |
| 1000 | 1000 | 32.12 | 4.23 | 579.81 | 451.57 | 20.02 | 1554.52 | 472.71 | 61.28 | 16.99 | 27.8 |
| 1000 | 1500 | 35.67 | 4.83 | 756.94 | 716.51 | 21.50 | 1453.86 | 497.41 | 88.81 | 17.87 | 28.4 |
| 1000 | 2000 | 35.99 | 4.60 | 935.18 | 955.41 | 23.16 | 1513.09 | 505.07 | 114.69 | 17.61 | 27.1 |
| 1000 | 2500 | 36.00 | 4.18 | 1095.89 | 1225.99 | 20.32 | 1418.64 | 473.81 | 122.86 | 17.39 | 27.6 |
| 1000 | 3000 | 37.21 | 4.52 | 1308.67 | 1508.45 | 22.51 | 1505.15 | 509.36 | 156.23 | 20.11 | 27.1 |
| 1000 | 3500 | 38.67 | 4.90 | 1483.86 | 1829.46 | 22.24 | 1401.60 | 529.49 | 182.85 | 17.67 | 29.9 |
| 1000 | 4000 | 37.77 | 4.22 | 1663.13 | 2096.18 | 19.75 | 1472.58 | 482.78 | 203.06 | 16.85 | 28.4 |
| 1000 | 4500 | 41.22 | 4.95 | 1820.08 | 2419.59 | 20.83 | 1361.44 | 505.90 | 229.47 | 18.05 | 27.2 |
| 1000 | 5000 | 39.81 | 4.37 | 2370.21 | 2688.93 | 20.53 | 1390.98 | 502.21 | 238.62 | 17.91 | 27.9 |
| 1000 | 5500 | 41.70 | 4.87 | 2604.21 | 3027.27 | 21.45 | 1383.86 | 528.20 | 283.38 | 18.57 | 28.5 |
| 1000 | 6000 | 42.23 | 4.67 | 2803.79 | 3388.38 | 21.67 | 1446.13 | 501.96 | 316.17 | 18.01 | 26.6 |
| 1000 | 6500 | 42.46 | 4.99 | 3028.74 | 3645.43 | 21.50 | 1451.27 | 544.11 | 326.95 | 18.41 | 27.6 |
| 1000 | 7000 | 43.38 | 4.42 | 3211.73 | 3919.46 | 20.57 | 1423.18 | 521.60 | 329.16 | 17.40 | 27.1 |
| 1000 | 7500 | 45.91 | 4.48 | 3507.97 | 4291.63 | 23.89 | 1447.30 | 507.35 | 338.57 | 17.07 | 28.1 |
| 1000 | 8000 | 43.38 | 4.59 | 3656.90 | 4586.98 | 21.57 | 1417.73 | 511.91 | 364.30 | 18.13 | 28.3 |
| 1000 | 8500 | 46.58 | 05.03 | 3863.04 | 4916.73 | 21.40 | 1492.24 | 512.49 | 437.60 | 18.40 | 29.3 |
| 1000 | 9000 | 47.30 | 4.43 | 4042.96 | 5237.41 | 21.21 | 1424.07 | 526.02 | 497.90 | 18.12 | 27.0 |
| 1000 | 9500 | 46.68 | 4.43 | 4605.68 | 5490.45 | 20.37 | 1463.82 | 526.39 | 436.37 | 19.46 | 26.8 |
| 1000 | 10000 | 45.80 | 4.20 | 4889.18 | 5877.01 | 20.03 | 1485.12 | 534.18 | 464.18 | 17.52 | 27.2 |


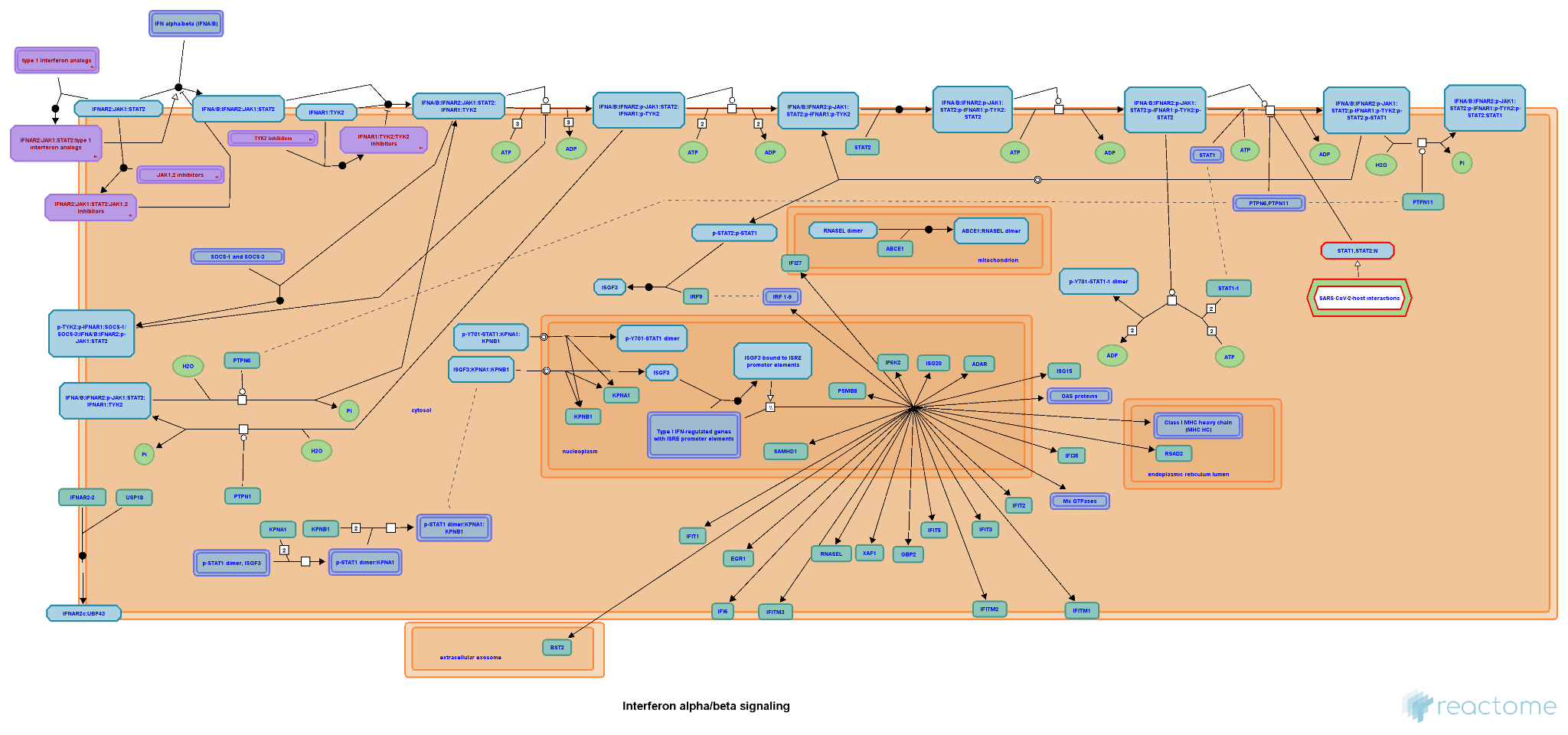


*Figure S1. Overview of the Reactome Interferon alpha/beta signaling pathway. Downloaded from Reactome Database with Reactome Viewer.*


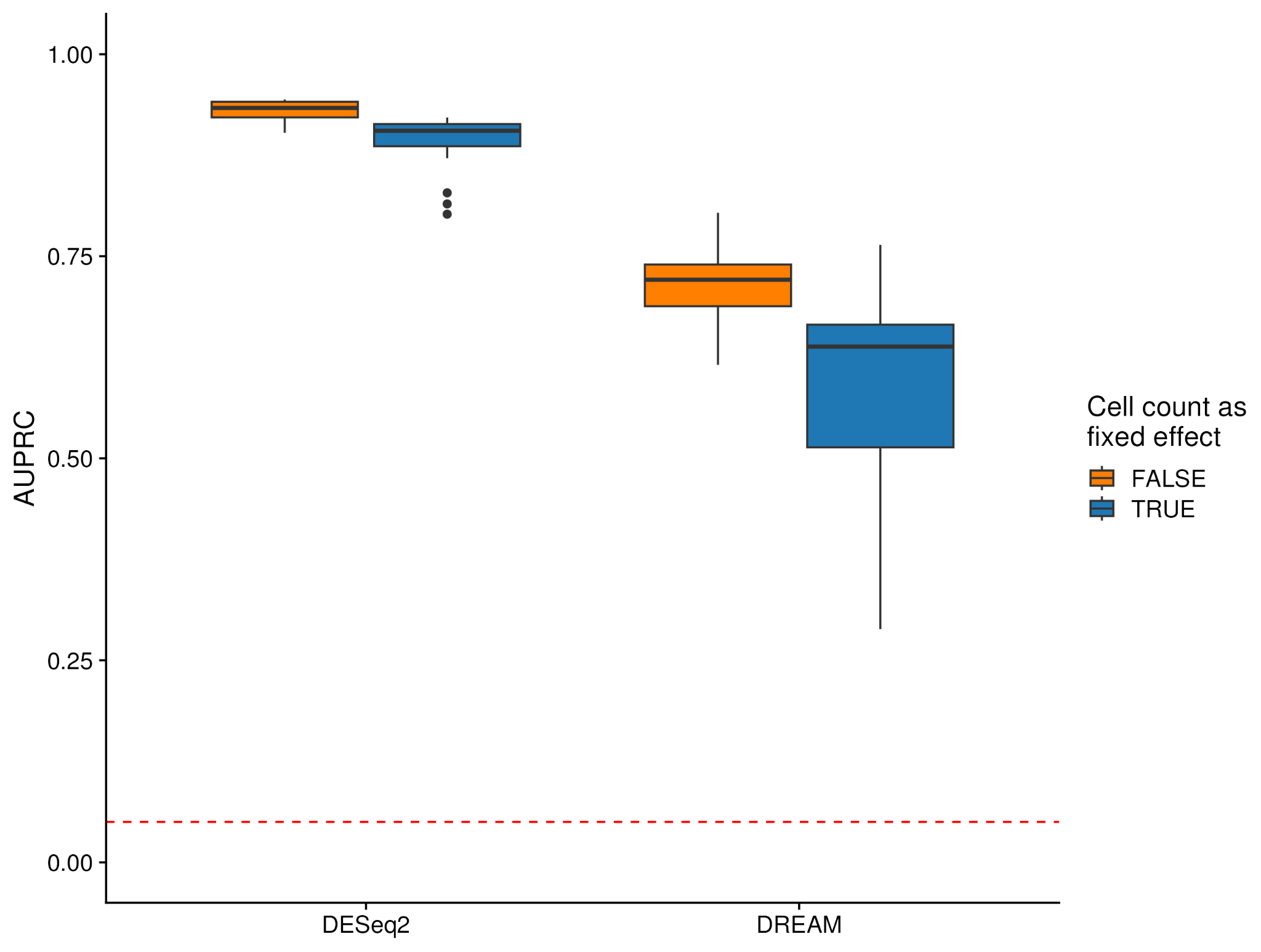


*Figure S2. Cell count per pseudobulk sample as fixed effect. The number of cells each pseudobulk consists of was incorporated as a fixed effect into the formula for the parametric pseudobulk methods DESeq2 and DREAM to check whether this increases the performance. The random baseline is indicated by the dashed line.*


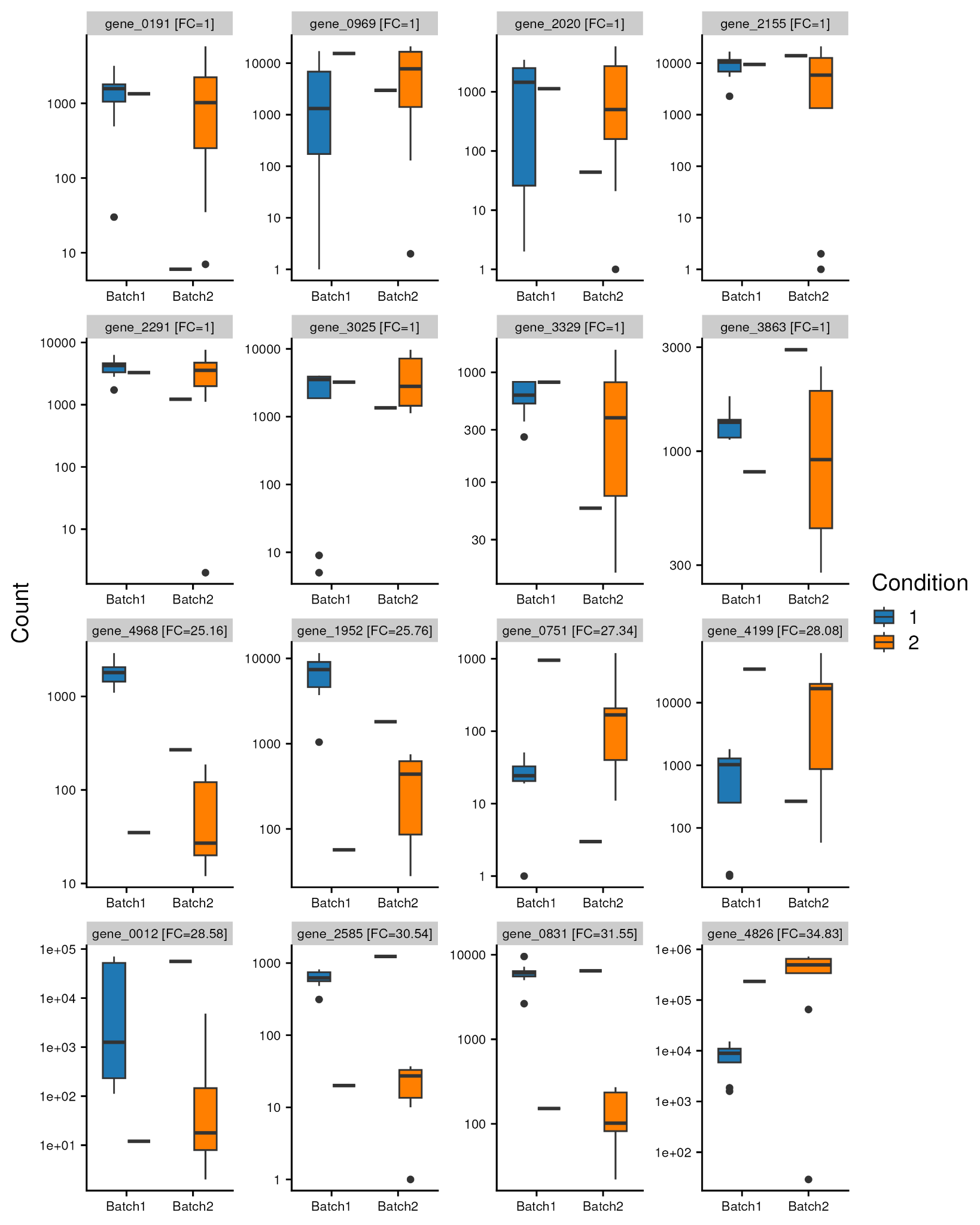


*Figure S3. Gene expression counts across batches and conditions in the unbalanced atlas setting. The figure shows the simulated expression per batch and condition for 8 randomly selected genes simulated not to be differentially expressed (log fold change = 0; top two rows) and the 8 genes with the highest differential expression by simulated log fold change (bottom two rows). The plot is based on the pseudobulked unbalanced atlas setting, each point refers to the gene count of one sample.*


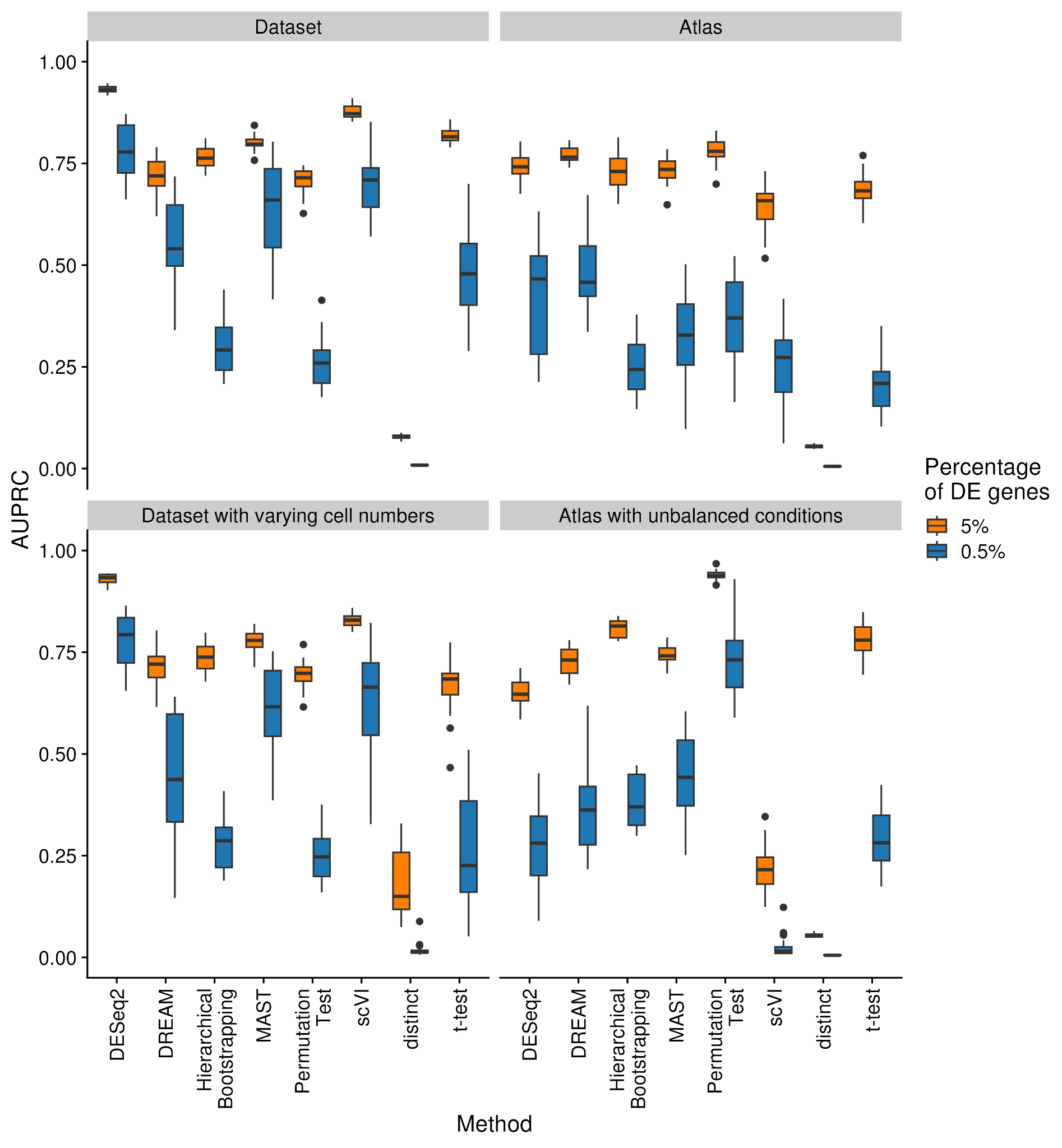


*Figure S4. Performance differences with varying DE gene percentage. Performance of all methods on all scenarios with different amounts (5% or 0.5%) of differential expressed genes. The scenarios refer to the four main simulation scenarios.*


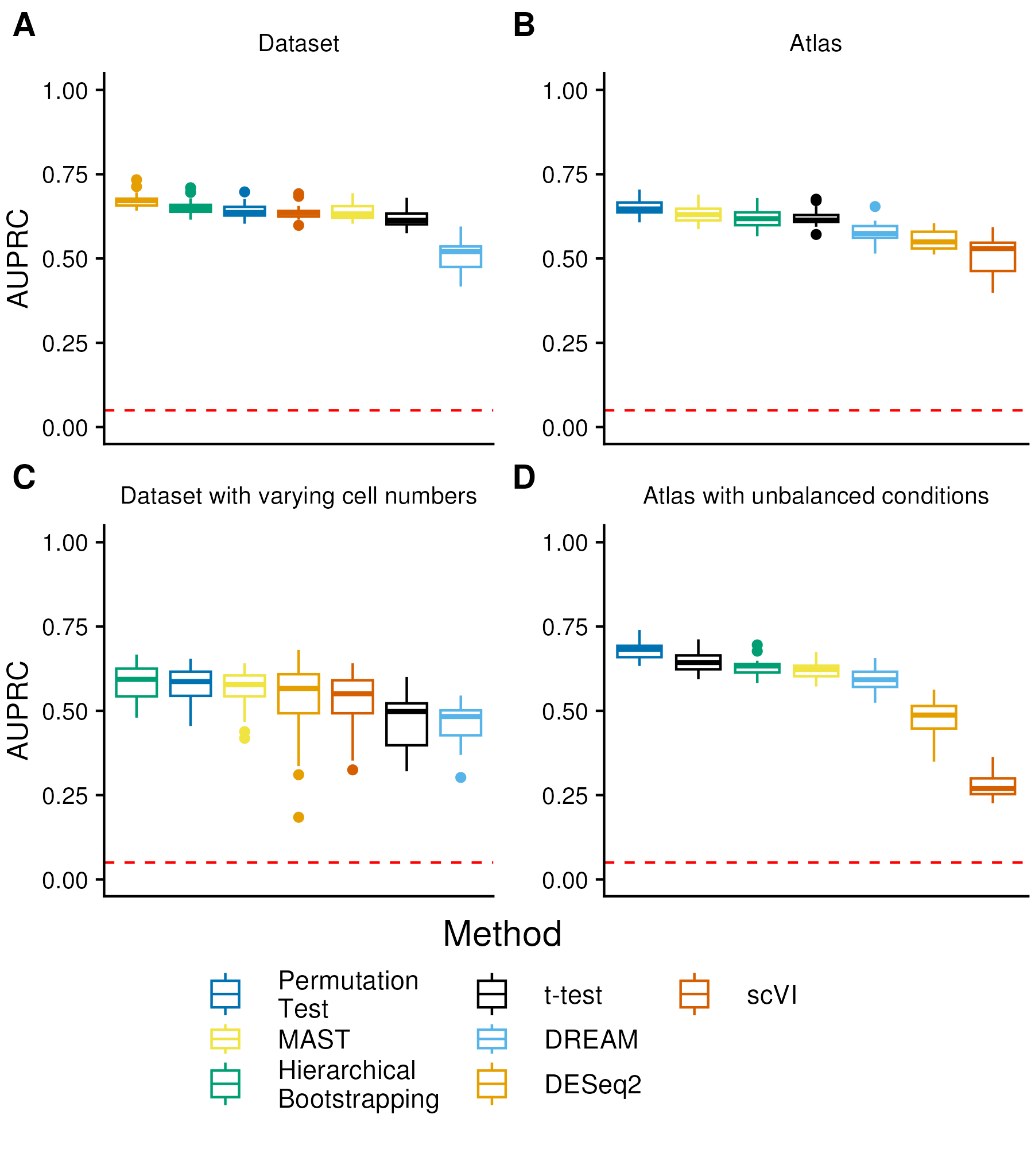


*Figure S5. Performance of the methods on the simulated scenarios after filtering for the top 10% of highly variable genes. Shown is the performance of the methods on the simulated scenarios over 20 independent simulations and runs each for (A) the Dataset scenario, (B) the Atlas scenario, (C) the dataset scenario with varying cell numbers per sample, and (D) the Atlas scenario with unbalanced conditions. Before the methods were applied to the simulated scenarios, the datasets were filtered for 10% of the highly variable genes. Removed differentially expressed genes are classified as false negatives. The methods were sorted based on their performance. The random baseline is indicated by the dashed line.*


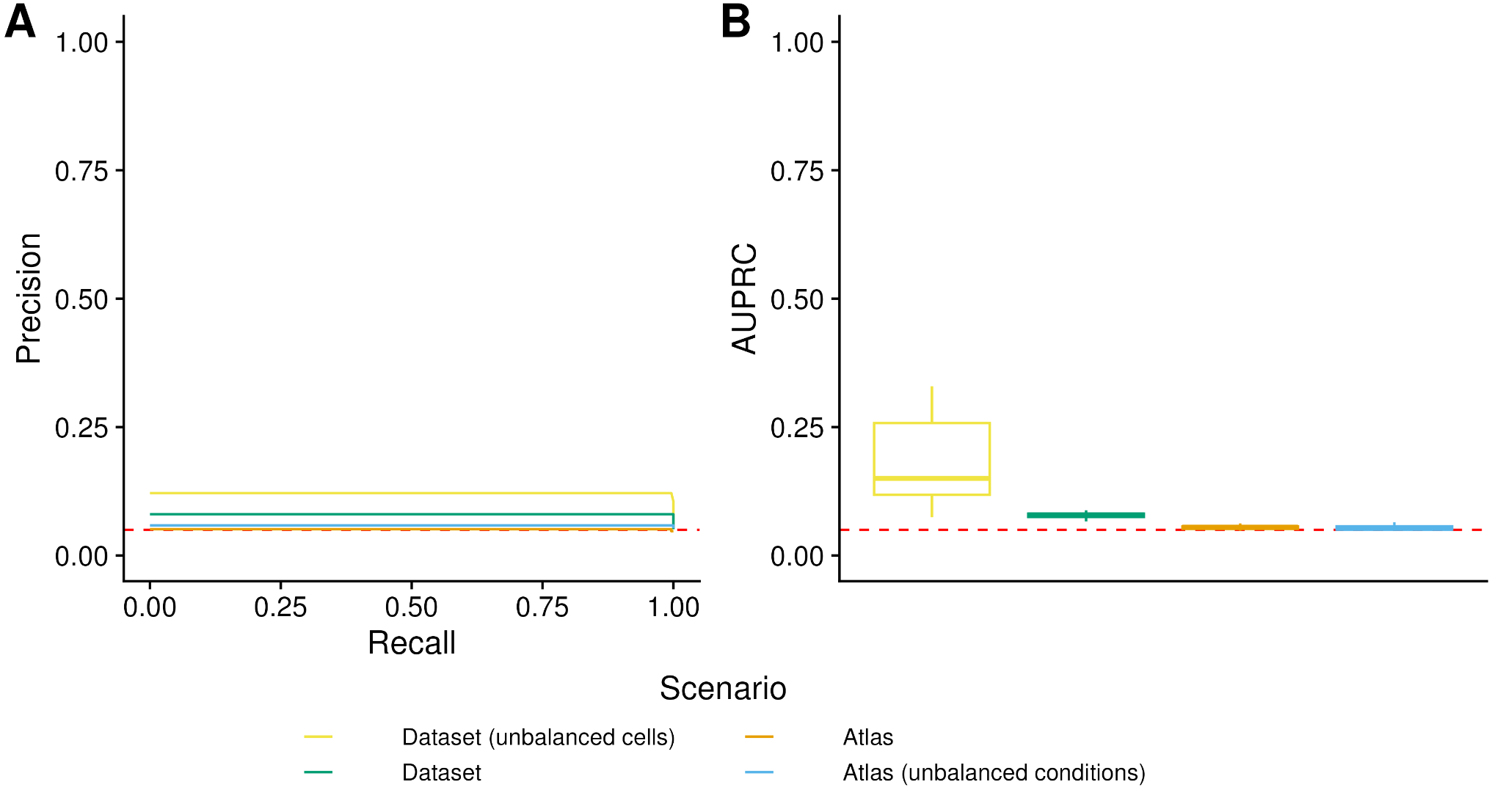


*Figure S6. ​​Performance of distinct on the simulated scenarios. Distinct showed baseline performance in almost all simulated scenarios and was therefore excluded from the main benchmark. It assigns the minimal p-value to the majority of the genes and which makes them hard to rank.*


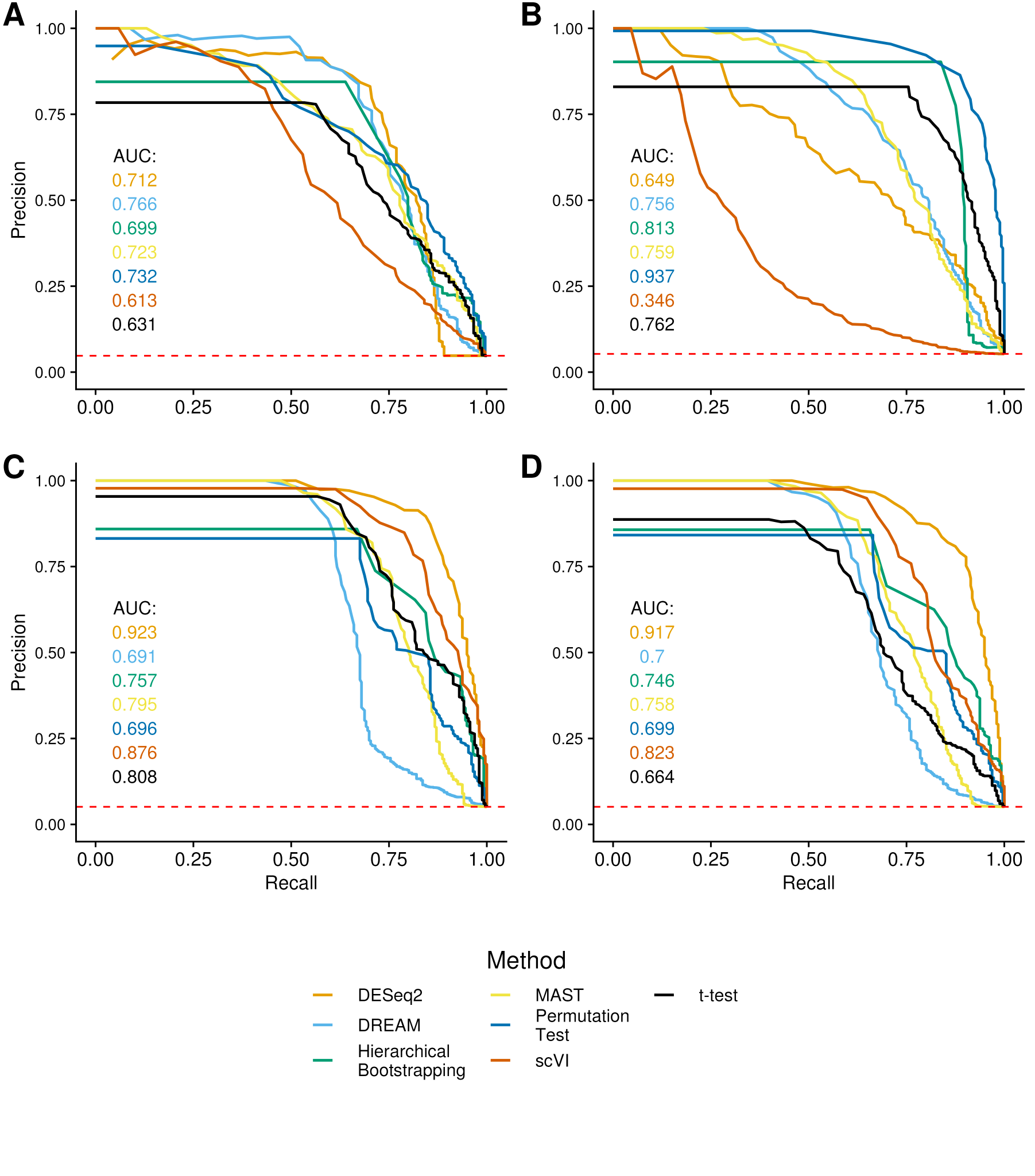


*Figure S7. Precision-recall plot for all scenarios on a single simulation. Shown are the precision-recall plots for one of the 20 simulations based on the predictions of all methods except distinct. (A) shows the data for the atlas scenario, (B) for the atlas scenario with unbalanced conditions, (C) for the dataset scenario and (D) for the dataset scenario with variable cell numbers.*


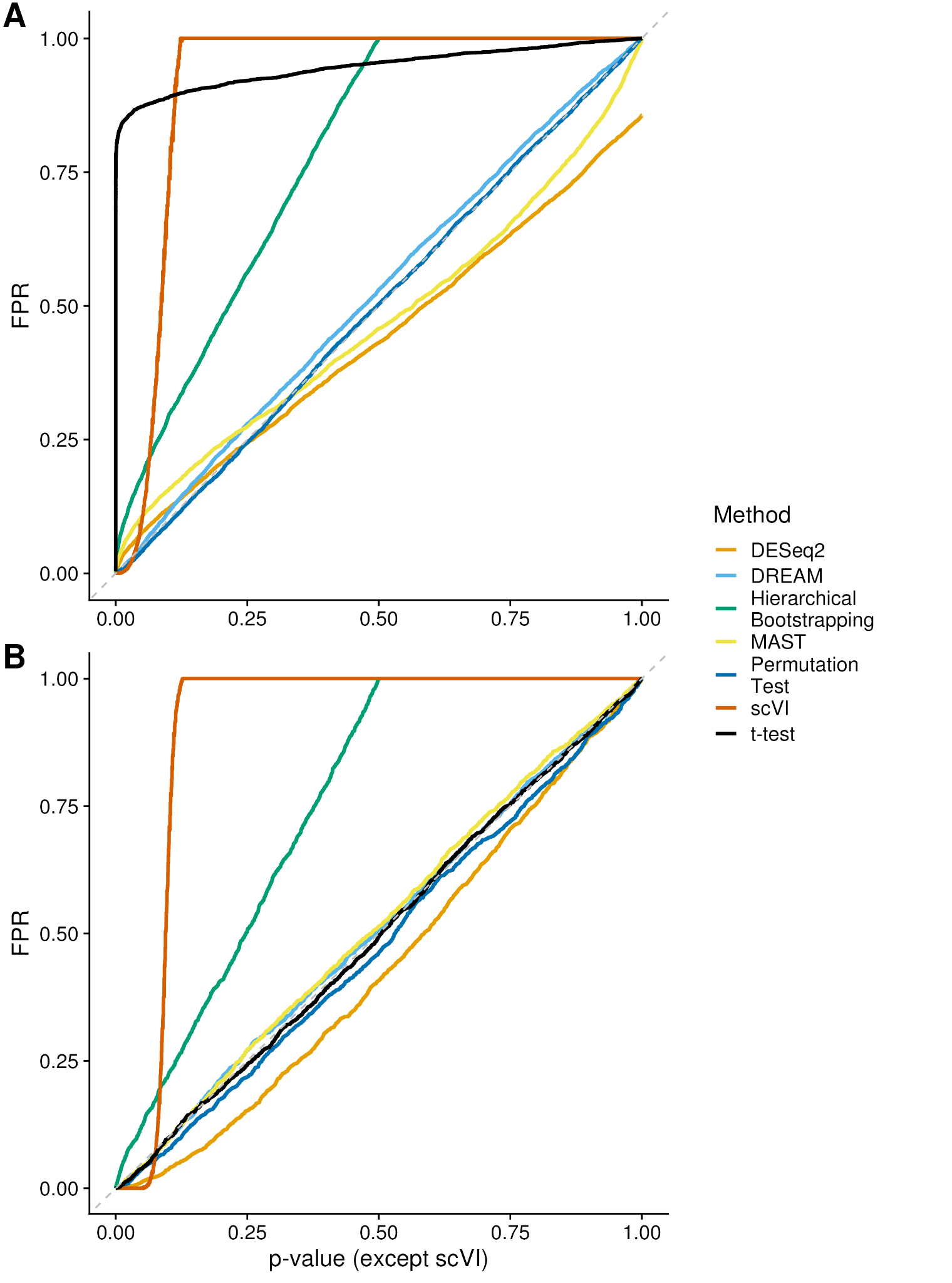


*Figure S8. Negative control based on raw p-values and false positive rate (FPR). Negative control based on (A) the simulated atlas scenario from Fig. 7 without zoom with no differential expression and (B) the real data scenario from the Seurat vignette. The real data has been permuted to eliminate differential expression across conditions. Since scVI does not generate traditional p-values but instead uses a Bayesian decision rule to determine differential expression, we assessed its classification results across 2000 equidistant cutoffs between 0 and 1, calculating false positives from these outcomes.*


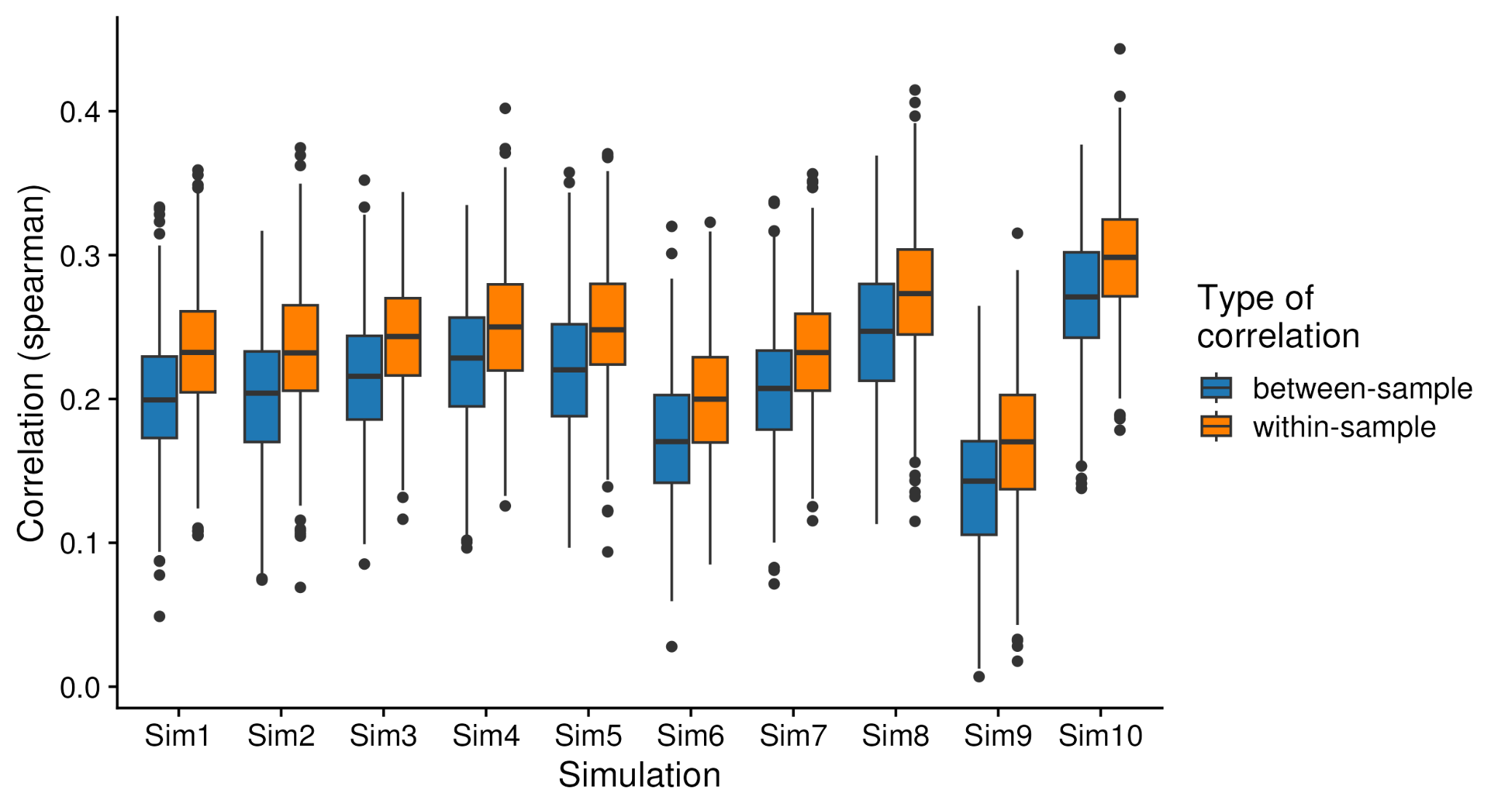


*Figure S9. Within- and between-sample Spearman correlations on the dataset scenario.The correlations are calculated based on the expression of two cells and depict the similarity of the expression of these cells to each other. The median of the correlations within a sample is always larger than the median across samples and therefore indicates a higher similarity of cells within a sample. The middle line represents the median, and the lower and upper limits of the boxes specify the 25% quartile and the 75% quartile. The whiskers extend to the largest/smallest correlation point, within 1.5 × interquartile range. Method adapted from Ref. [1]*

*
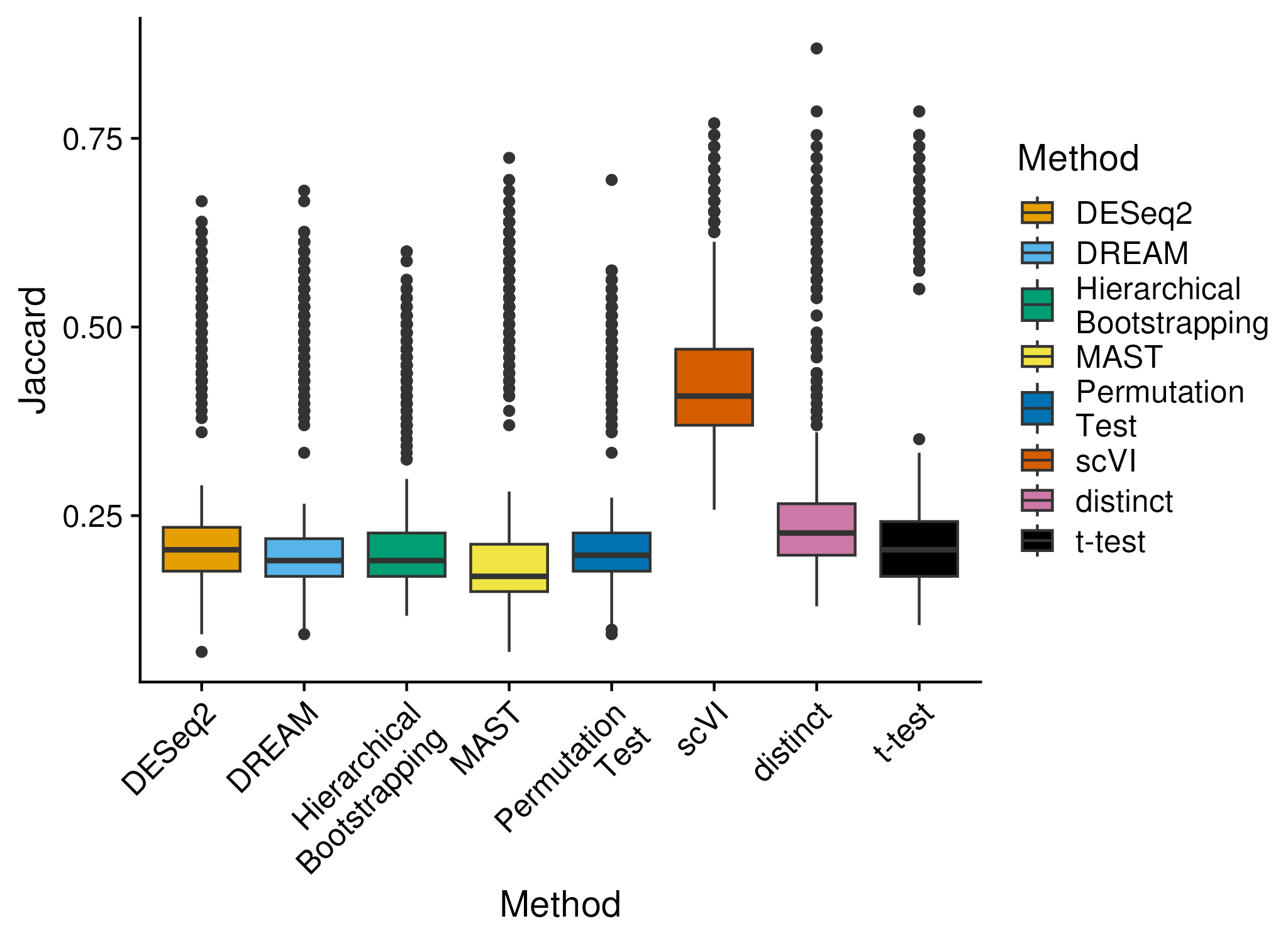
*

*Figure S10. Boxplot showing the distribution of pairwise Jaccard indices calculated for the top 100 genes with the smallest p-values. The analysis was conducted on 42 subsets of the lung cancer atlas [27], comparing samples from lung adenocarcinoma and lung squamous cell carcinoma. Differential expression methods were applied to each subset, and the Jaccard index was computed to measure the overlap of the top 100 genes across all possible pairs of subsets. This visualization summarizes the consistency of gene selection across subsets and methods.*

*
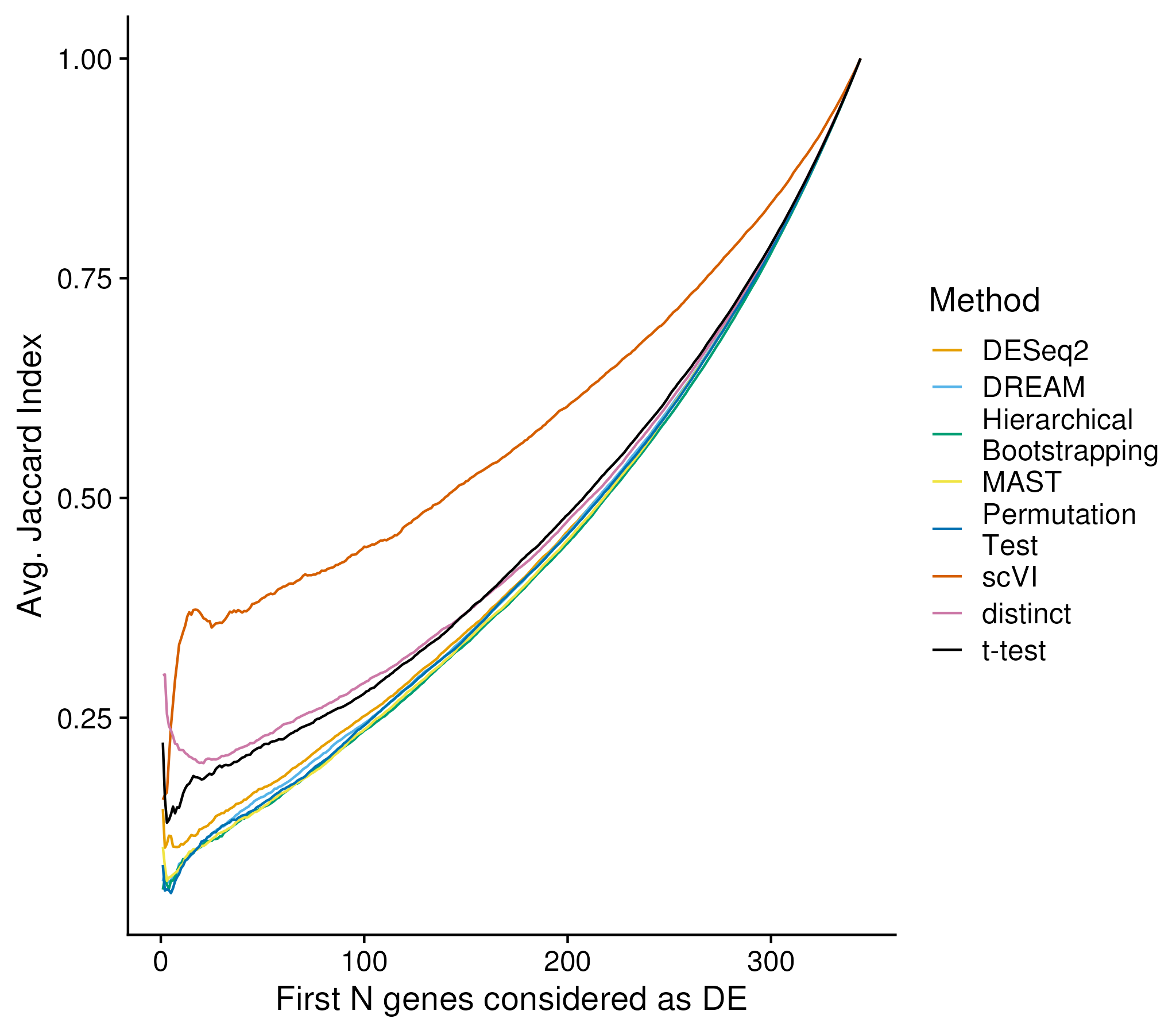
*

*Figure S11. Line plot showing the average Jaccard index across all pairwise comparisons of the 42 subsets from Fig. S10, calculated for varying numbers of top genes (N) ranked by smallest p-values. The x-axis represents N, the number of genes considered, and the y-axis shows the average Jaccard index, highlighting how the overlap between subsets changes as more genes are included in the comparison.*

*
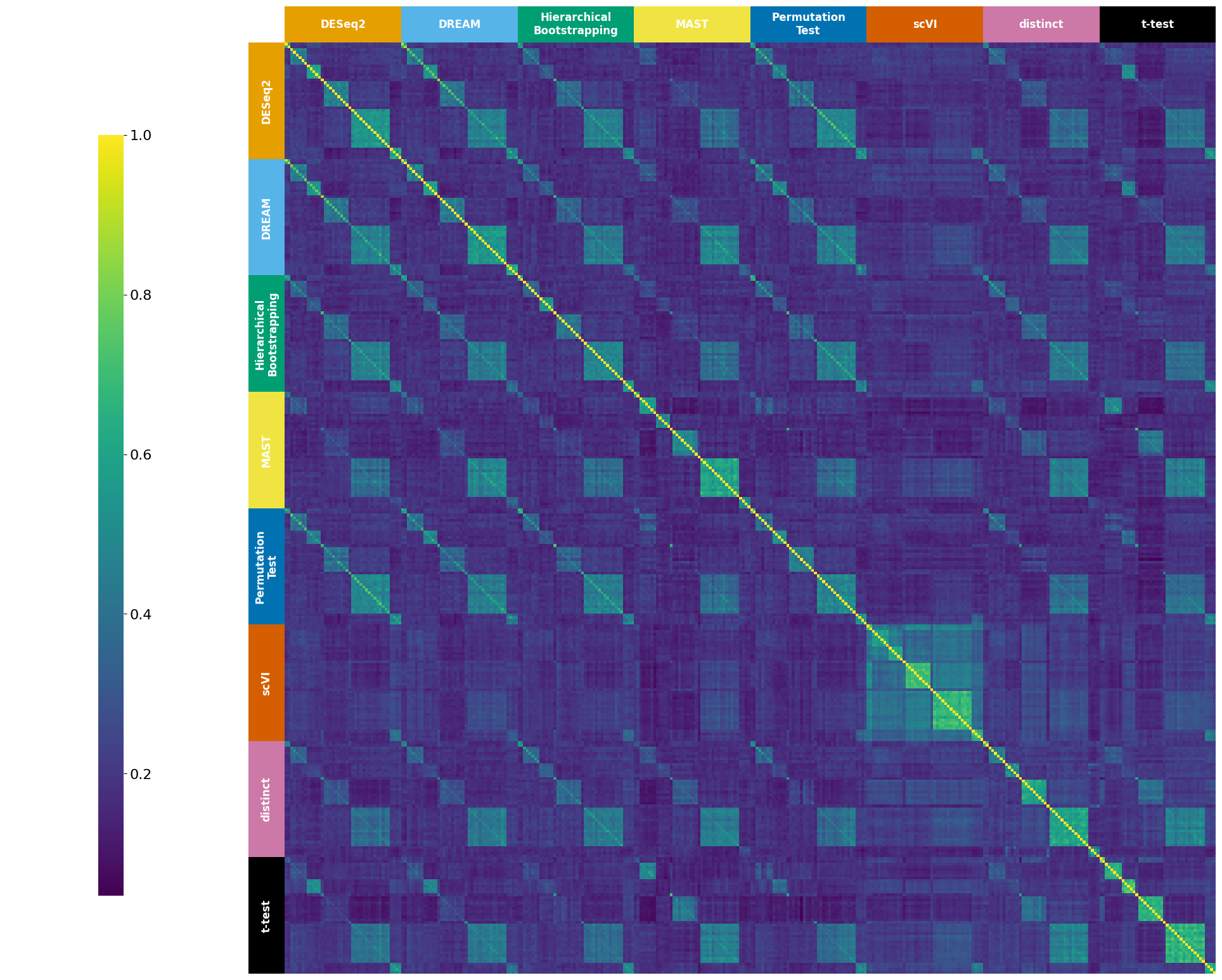
*

*Figure S12. Heatmap displaying the pairwise Jaccard indices for the top 100 genes with the smallest p-values, computed across all methods and subsets of the lung cancer atlas [27]. The x-axis and y-axis represent all methods, with 42 columns and rows for each method corresponding to the 42 subsets. Each cell shows the Jaccard index between a pair of methods and subsets, with the color intensity indicating the degree of overlap. This visualization highlights both within-method and between-method consistency in gene selection across subsets.*
